# Efficient generation of mutations mediated by CRISPR/Cas9 in the hairy root transformation system of *Brassica carinata*

**DOI:** 10.1371/journal.pone.0185429

**Published:** 2017-09-22

**Authors:** Thomas W. Kirchner, Markus Niehaus, Thomas Debener, Manfred K. Schenk, Marco Herde

**Affiliations:** 1 Institute of Plant Nutrition, Leibniz Universitaet Hannover, Hannover, Germany; 2 Institute for Plant Genetics, Leibniz Universitaet Hannover, Hannover, Germany; Agriculture and Agri-Food Canada, CANADA

## Abstract

A protocol for the induction of site-directed deletions and insertions in the genome of *Brassica carinata* with CRISPR is described. The construct containing the Cas9 nuclease and the guide RNA (gRNA) was delivered by the hairy root transformation technique, and a successful transformation was monitored by GFP fluorescence. PAGE analysis of an amplified region, presumably containing the deletions and insertions, demonstrated up to seven different indels in one transgenic root and in all analyzed roots a wildtype allele of the modified gene was not detectable. Interestingly, many of these mutations consisted of relatively large indels with up to 112 bp. The exact size of the deletions was determined to allow an estimation whether the targeted gene was not functional due to a considerable deletion or a frame shift within the open reading frame. This allowed a direct phenotypic assessment of the previously characterized roots and, in fact, deletions in *FASCICLIN-LIKE ARABINOGALACTAN PROTEIN 1* (*BcFLA1*)–a gene with an expression pattern consistent with a role in root hair architecture–resulted in shorter root hairs compared to control roots ectopically expressing an allele of the gene that cannot be targeted by the gRNA in parallel to the CRISPR construct. As an additional line of evidence, we monitored *BcFLA1* expression with qPCR and detected a significant reduction of the transcript in roots with an active CRISPR construct compared to the control, although residual amounts of the transcript were detected, possibly due to inefficient nonsense-mediated mRNA decay. Additionally, the presence of deletions and insertions were verified by Sanger sequencing of the respective amplicons. In summary we demonstrate the successful application of CRISPR/Cas9 in hairy roots of *B*. *carinata*, the proof of its effectiveness and its effect on the root hair phenotype. This study paves the way for experimental strategies involving the phenotypic assessment of gene lesions by CRISPR which do not require germline transmission.

## Introduction

With the advent of next-generation sequencing, the scientific community was provided with a cost-effective approach of obtaining genome and transcriptome sequences of many non-model plants with unique features or high relevance for agriculture. Until recently, for a functional characterization of genes with a loss-of-function approach in non-model plants, that, for example, do not have a collection of insertional mutants, the available toolbox mainly consisted of the RNA interference (RNAi) technology [1].

The discovery of nucleases that induce double strand breaks at defined positions in the genome, resulting in site-directed deletions within a gene, was a major breakthrough, especially in plants where the targeted knockout of genes was challenging [2,3]. Notably, the clustered regularly interspaced short palindromic repeats (CRISPR) and the transcription activator-like effector nuclease (TALEN) technologies are easily available and applicable in all laboratories with standard equipment for molecular biology. In contrast to the previously used RNAi technique, the advantage of the TALEN/CRISPR approach is to completely abolish gene functionality and a lower off-target rate [4]. In recent years, the CRISPR/Cas9 system grew into a simple, well-established and efficient method for performing gene engineering in plants. In this system, the Cas9 nuclease is targeted to specific genomic sequences, where it induces double-strand breaks (DSBs). In somatic plant cells, these are predominantly repaired by non-homologous end joining (NHEJ), which leads to errors during the repair causing insertions, deletions (indels) or replacements [5]. The specificity of the CRISPR/Cas9 system, which was shown to be especially high in plants [6,7], is ensured by a 20 bp protospacer sequence belonging to the so-called guide RNA (gRNA), which guides the Cas9 nuclease to the corresponding site in the genomic DNA (gDNA). The protospacer sequence has to be complementary to the target sequence, which must be followed by the protospacer adjacent motif (PAM), consisting of e.g. ‘NGG’ so that the Cas9 can bind and cut the DNA double strand. However, base pairing of defined bases near the PAM, known as ‘seed sequence’, is essential and therefore decisive for the specificity [8–11] (for a review, see for example [12,13]).

However, a major bottleneck in the functional characterization of genes in non-model plants is the lack of fast and efficient transformation protocols for recalcitrant genotypes or whole species. Nonetheless, techniques such as *Agrobacterium* infiltration, protoplast transformation or hairy root transformation were developed which do not primarily aim at a transgenic event that enters the germline rather, they aim to transform a cell or organ whose features as a consequence of the transformed construct can be directly studied. These techniques are frequently used to evaluate the efficiency of the customized nuclease system such as the gRNAs for CRISPR [14] or the establishment of CRISPR in a certain plant species [15,16] and few studies used the hairy root technology in combination with CRISPR to directly gain insight into the gene function [17,18].

Since our research focus is on alteration of the root architecture in response to phosphate starvation, we used the *Agrobacterium rhizogenes*-mediated hairy root transformation technique [19] for delivery of the CRISPR construct. This approach offers the unique opportunity to swiftly examine the effects of a potential gene modification because these are directly visible in the hairy roots.

One of the challenges of such an approach is the necessity to assess the CRISPR-induced deletions quantitatively and qualitatively in every single root that is used for phenotyping, assuming that the deletions are heterogeneous amongst different transformation events. It must be ensured that the CRISPR mechanism was efficient in the actual tissue so that the wildtype allele is more or less completely absent. Furthermore, it is paramount to determine the exact number of deleted or inserted bases, as small in-frame codon deletions may not lead to a completely abolished gene function.

In this study, we explored the possibility of correlating deletions in a gene induced by a CRISPR construct delivered by hairy root transformation with a root hair phenotype. As a proof of principle, we chose *FASCICLIN-LIKE ARABINOGALACTAN PROTEIN 1* (*BcFLA1*), a gene in *Brassica carinata*, whose RNA-seq expression profile in a preliminary study suggested a role in the regulation of root hair elongation. The frequency of alleles with deletions in *BcFLA1* was sufficient to select roots that were presumably without a functional gene of interest and these roots were phenotypically altered compared to roots that were additionally complemented with a CRISPR resistant allele of the gene. We present a cost-effective, medium throughput approach to estimate the frequency of deletions, the absence of the wildtype allele as well as the deletion sizes in a single experiment. This approach, based on polyacrylamide gel electrophoresis, may complement the existing methods for the detection of genome indels and is uniquely suited for the evaluation of CRISPR-induced events in combination with the direct assessment of a putatively associated phenotype.

## Materials and methods

### Obtainment of *BcFLA1*

*BcFLA1* belongs to a list of candidate genes for the Pi deficiency-induced root hair elongation, which was obtained by a genome-wide expression profile analysis by massive analysis of cDNA ends (MACE; GenXPro GmbH, Frankfurt Main, Germany) [20].

The *BcFLA1* sequence obtained by MACE was extended by 5’ RNA ligase-mediated rapid amplification of cDNA ends (5’ RLM-RACE) using the ‘FirstChoice^®^ RLM-RACE Kit’ (Thermo Fisher Scientific, Waltham, MA, USA) according to the manufacturer´s instructions. For both outer and inner PCR, the KAPA HiFi HotStart PCR Kit (KAPA Biosystems, Wilmington, MA, USA) was applied according to the manufacturer’s specifications, in which ‘CAATCCATGCATCATATCCAAC’ was used as the outer and ‘ACCCAAATCAAACGACGAGT’ as the inner reverse primer. The products were cloned into the pJET1.2/blunt vector (Thermo Fisher Scientific, Waltham, MA, USA) and sequenced by GATC Biotech AG (Konstanz, Germany) and SEQLAB (Göttingen, Germany), respectively. During validation of the CRISPR/Cas9-induced gene editing, we observed a second allele of *BcFLA1* with a similarity of 94%. The cDNA and gDNA sequences of both the first (*BcFLA1a*) and the second allele (*BcFLA1b*) were submitted to the NCBI database (https://www.ncbi.nlm.nih.gov) with the accession numbers KY905141 for *BcFLA1a* (cDNA) and KY905142 for *BcFLA1b* (cDNA) as well as KY965932 for *BcFLA1a* (gDNA) and KY965933 for *BcFLA1b* (gDNA).

### Construct preparation

#### gRNA design

The *BcFLA1* gene sequence was scanned for PAM sequences (NGG) on both gDNA strands. A list of potential gRNAs was filtered in consideration of the following quality standards: GC content at least 50% [21], gRNAs had to be located within an exon, not more than six double-stranded bases in a row within the protospacer´s secondary structure [21] predicted by ‘The mfold Web Server’ [22] and the gRNAs should be as near as possible at the 5’ end of the *BcFLA1* coding sequence. Two different gRNAs were selected within a distance of 69 bp (gR1: CTGTCTTCCTCTCCACGGAG, gR2: ACATCACGGCGATCCTTGAA). To enable the usage of both gRNAs within one construct, a polycistronic tRNA-gRNA gene was cloned as described previously [23] with the following changes: the restriction digestion of the tRNA-gRNA gene and the vector was done using Anza^TM^ BpiI (Thermo Fisher Scientific, Waltham, MA, USA) according to the manufacturer’s protocol, following an insertion of the tRNA-gRNA gene into pB-CRISPR+35S::GFP (V112) vector *via* the BpiI cutting site creating pB-CRISPR+35S::GFP+fla1-guides (H278) (S1A Fig), so that the primer sequences had to be adjusted (S1 Table). Furthermore, the PCR products were purified using the ‘Fast Gene Gel/PCR Extraction Kit’ (Nippon Genetics Europe, Dueren, Germany) and the PCR using the ligation product as template was done using the Phusion^®^ High-Fidelity DNA Polymerase (New England Biolabs, Ipswich, MA, USA) as in the first PCR.

#### Vectors

CRISPR+35S::GFP (V112) was constructed (S1 Text) to possess a GFP cassette for the selection of the transgenic hairy roots containing a GFP gene with introns for reduced silencing under control of a 35S promotor. Furthermore, it consisted of a CRISPR cassette containing the Cas9 gene driven by the ubiquitin4-2 promoter from parsley and the polycistronic gene carrying the two gRNAs under control of the U6-26 promoter (S1A Fig).

A complementation vector (H280) was constructed (S1 Text) from pB-CRISPR+35S::GFP+fla1-guides additionally possessing an overexpression cassette, containing the CDS of a mutated *BcFLA1* (*BcFLA1a*_*mut*_) enclosed by the same artificial UTRs used for the Cas9 and under control of the ubiquitin4-2 promoter (S1B Fig). The mutation consisted of three base pairs (bp) in each of the gRNA target sequences, which were exchanged codon-optimized according to the codon usage in *Arabidopsis thaliana* without changing the amino acid sequence (S1C Fig). One of the exchanges destroyed the PAM sequence of both gRNAs so that the Cas9 was unable to bind and cut these sites. Therefore, *BcFLA1a*_*mut*_ was resistant against the CRISPR mechanism in combination with the two gRNAs used in this study.

As a control, pB-CRISPR+35S::GFP (V112) vector without gRNAs was introduced into the plants.

The completed plasmids were transformed into electrocompetent *E*. *coli* DH10B or DH5α, confirmed by Sanger sequencing (GATC Biotech AG, Konstanz, Germany) and then transformed into electrocompetent *Agrobacterium tumefaciens* C58, containing a root-inducing (Ri) plasmid (ARqua1).

### Plant material, cultivation and hairy root transformation

We chose *B*. *carinata*, because varieties of this species were shown to differ in their response to P and N deficiency regarding the root hair formation. Seeds of *B*. *carinata* cv. Bale were vernalized for three days in the dark at 4°C. and then germinated for 3 days (photoperiod, 16/8 h light/dark; temperature, 18/15°C day/night; relative humidity, 75%; light intensity, 220 μmol m^-2^ s^-1^). Hairy roots were induced by following a modified protocol from [24]. *Agrobacterium tumefaciens* C58 (ARqua1) was grown for two days on a YEB plate, scratched off the plate and resuspended in 6 mL PS buffer (0.7% Na_2_HPO_4_ (w/v), 0.3% KH_2_PO_4_ (w/v), 0.5% NaCl (w/v), 150 μM acetosyringone, pH 7). After an incubation time of at least 1.5 h, the *B*. *carinata* seedlings were dampened with the bacterial solution and wounded along the hypocotyl with an insulin syringe (U-40 Insulin, 0.3 mm x 12 mm). Additionally, the bacterial solution was injected into the plants. Then, the seedlings were further cultivated in clay granulate under the same conditions as during the germination, except for an incubation in the dark for 20 h directly after the transformation. After one week, the plants were fertilized with a nutrient solution (pH 5.3) containing (mM) 2.25 Ca(NO_3_)_2_ x 4 H_2_O, 2.5 K_2_SO_4_, 1 MgSO_4_ x 7 H_2_O, 0.25 KCl, 1 KH_2_PO_4_ and (μM) 25 H_3_BO_3_, 1.5 MnSO_4_, 1.5 ZnSO_4_, 0.5 CuSO_4_, 0.025 (NH_4_)_6_Mo_7_O_24_ and 35.8 Fe (Fe^III^-EDTA). Two weeks after the transformation, clay granulate was earthed up to cover the grown hairy roots. One additional week later, the plants were screened for transgenic roots using GFP as fluorescent marker (‘SMZ25’, Nikon, Düsseldorf, Germany, or ‘FastGene^®^ BG-LED Flashlight’, Nippon Genetics Europe, Dueren, Germany). The non-transgenic roots were cut off and plants with at least one transgenic root were further cultivated for eight days under the same conditions as before and in the same nutrient solution which was used as fertilizer, but without KH_2_PO_4_ and pH was readjusted to 5.3 during the cultivation as needed. Then, 2-cm root tips were harvested for root hair length measurement as well as RNA and gDNA extraction.

### RNA & gDNA isolation

RNA and gDNA were isolated using the peqGOLD TriFast™ reagent according to the manufacturer’s instructions (VWR International GmbH, Darmstadt). For RACE-PCR, RNA was isolated using the ‘NucleoSpin^®^ miRNA’ Kit (Machery-Nagel, Dueren, Germany) following the manufacturer’s specifications (using the total RNA isolation procedure). RNA Quality was electrophoretically tested on a 1% (w/v) non-denaturating agarose gel containing 0.004% (v/v) Midori Green Advance (Nippon Genetics Europe, Dueren, Germany) for a runtime of 35 min at 7.6 V cm^-1^ in Tris-acetate-EDTA (TAE) buffer. Both RNA and gDNA were quantified using the NanoPhotometer^®^ P-Class P 300 (Implen, Munich, Germany).

### Expression analysis

Wildtype- and *BcFLA1a*_*mut*_-specific primers were designed on the differing bases in the gRNA binding sites. Additionally, unspecific primers for the total *BcFLA1* expression were designed with binding sites near the 3’ end of *BcFLA1*.

The geometric mean of the Ct values of two endogenous control genes was calculated according to Vandesompele *et al*. [25], which was then used to calculate the fold over reference of the different samples (2^-delta Ct^).

For more detailed information regarding the cDNA synthesis, primer design, primer efficiency tests, endogenous controls and quantitative PCR (qPCR) conditions we refer to the S2 Text.

### Verification of the gene editing

For the verification of the gene editing, the gene region including the two gRNA regions was amplified. For it, *BcFLA1a*_*mut*_ -specific, wildtype-specific and unspecific primers were designed using Primer3Plus [26] in consideration of the following quality standards: amplicon size between 600 and 800 (large) or 200 and 260 (small) bp, primer size between 20 and 25 bp, primer T_m_ between 58 and 62°C and a GC content between 40 and 60% (S3 Table). The specificity of the primers was realized by placing at least one primer into the wildtype or artificial UTRs and tested in the same way as the qPCR primers, whereupon the large amplicons were separated on a 2.5% (w/v) agarose gel for 45 min at 6.3 V cm^-1^.

The principle of the M13-SSR-PCR was described previously [27]. A M13-tail (GTAAAACGACGGCCAGT) was added to the 5’ end of the forward primers and a nested PCR was performed with each 10 μL reaction containing: 1x B1 buffer, 2 pmol dNTPs, 0.25 pmol M13 tailed forward *BcFLA1* (wildtype-spec. small) primer, 1.25 pmol 700 infrared dye (IRD) labelled M13 forward primer, 2.5 pmol reverse *BcFLA1* (wildtype-spec. small) primer, 0.3 U ‘DCS DNA HotStart Polymerase’ (DNA Cloning Service e.K., Hamburg, Germany) and 20 ng template. The protocol consisted of an initial step of 95°C for 10 min, followed by 24 cycles of 95°C for 1 min, 60°C or 63°C for 1 min and 72°C for 1 min, as well as 8 cycles of 95°C for 1 min, 52°C for 45 sec and 72°C for 1 min, and a final step of 72°C for 10 min.

A 500 bp sequence was synthesized (Integrated DNA Technologies, Inc., Coralville, IA, USA) without the same nucleotides occurring consecutively, whereby the beginning and end of the sequence matched the *BcFLA1* (unspec. large) primers. The latter were then used in a M13-SSR-PCR as described before and the resulting fluorescently labelled amplicon served as a template for a sequencing reaction by the “Thermo Sequenase Cycle Sequencing Kit” (Thermo Fisher Scientific, Waltham, MA, USA). The fluorescently labelled fragments were used as ladder with 1 bp intervals. The sequencing reaction was done according to the manufacturer´s instructions but, a formamide loading dye consisting of 98% formamide, 10 mM EDTA (pH 8) and 0.05% pararosaniline was used instead of the stop solution.

A 4% polyacrylamide gel with a thickness of 0.25 mm, containing 7.5 M urea, was prepared using the Rotiphorese^®^ DNA Sequencing System PK 1 (Carl Roth GmbH + Co. KG, Karlsruhe, Germany) according to the manufacturer’s instructions. Formamide loading dye was added to the samples following a denaturation step at 95°C for 6 min. The separation was performed using the 4300 DNA Analyzer (LI-COR, Lincoln, NE, USA) in Tris-borate-EDTA (TBE) buffer at 1500 V, 40 mA, 40 W, 45°C and a wavelength of 700 nm.

In addition to fragment analysis by polyacrylamide gel electrophoresis (PAGE), the amplicons were separated by agarose gel electrophoresis. For the first experiment, a nested PCR was performed as described for the PAGE analysis, except for the cycle numbers, which were increased to 26 and 10 respectively. For the replicated experiment, PCR was performed with *BcFLA1* (unspec. large) primers using the KAPA HiFi HotStart PCR Kit (KAPA Biosystems, Wilmington, MA, USA) together with the supplied GC buffer, 50 ng of template, a primer concentration of 0.3 μM and a cycle number of 35 according to the manufacturer’s instructions. The products were separated on a 2.5% (w/v) non-denaturating agarose gel containing 0.004% (v/v) Midori Green Advance (Nippon Genetics Europe, Dueren, Germany) for 3.75 h at 4.1 V cm^-1^ in TAE buffer. All visible bands were cut out of the gel and DNA was purified using the ‘PCR clean-up Gel extraction’ kit (Machery-Nagel, Dueren, Germany) according to the manufacturer’s instructions. Purified DNA was additionally amplified (only replication 1) and sequenced by GATC Biotech AG (Konstanz, Germany) or SEQLAB (Göttingen, Germany), using the *BcFLA1* (wildtype-spec. small) (replication 1) and *BcFLA1* (unspec. large) (replication 2) reverse primer.

### Root hair length measurement

2-cm root tips were fixed in 70% (v/v) EtOH overnight, stained in 0.05% (w/v) Toluidine Blue for 3 h and stored in tap water at 4°C. For root hair length measurement, root tips were pulled over 0.3% (w/v) agar-agar until the root hairs were straight. Pictures were taken using the ‘SMZ25’ (Nikon, Düsseldorf, Germany) and root hair length was measured using the corresponding software ‘NIS-Elements’. The measurement was done in a region with the longest and fully developed root hairs of 2-cm root tips cut from the transgenic first and second order lateral roots.

## Results

### Proof of concept study with *BcFLA1*

The background to this work was the study of the phosphate deficiency-induced root hair elongation in the non-model organism *Brassica carinata*, the Ethiopian mustard. In our previous studies, we observed that enhanced root hair length was paralleled by increased expression of *BcFLA1*, indicating a role in the regulation of the root hair elongation. *BcFLA1* is a member of the arabinogalactan protein (AGP) family that is *inter alia* thought to have regulative functions and AGPs possessing fasciclin (FAS) domains are predicted to be involved in protein-protein interactions [28]. Thus, we hypothesized root hair length in roots with a *BcFLA1* loss-of-function is reduced.

We customized a CRISPR vector with a strong ubiquitously active promoter (ubiquitin promoter from parsley) driving Cas9 expression and the U6-26 promoter from Arabidopsis controlling the expression of the gRNA (S1A Fig). The vector can be easily modified in one step to target any gene of interest with target-specific gRNAs. For *BcFLA1* two gRNAs with target sites at a distance of 69 bp (S1C Fig) were expressed in parallel using a polycistronic gRNA as described previously [23].

Since we cannot exclude that the gRNA used to target *BcFLA1* is able to produce off-target effects, especially in a non-model plant without a complete genome sequence, we added a mutated allele of *BcFLA1* (*BcFLA1a*_*mut*_) to the CRISPR vector as a control construct (called *BcFLA1* compl.) (S1B Fig). The mutations in the *BcFLA1* gene abolished binding of the gRNA but the wildtype amino acid sequence was maintained, allowing the ectopic expression of *BcFLA1* in the presence of the nuclease and gRNA (S1C Fig).

### Screening of the transgenic roots

**Fig 1:**
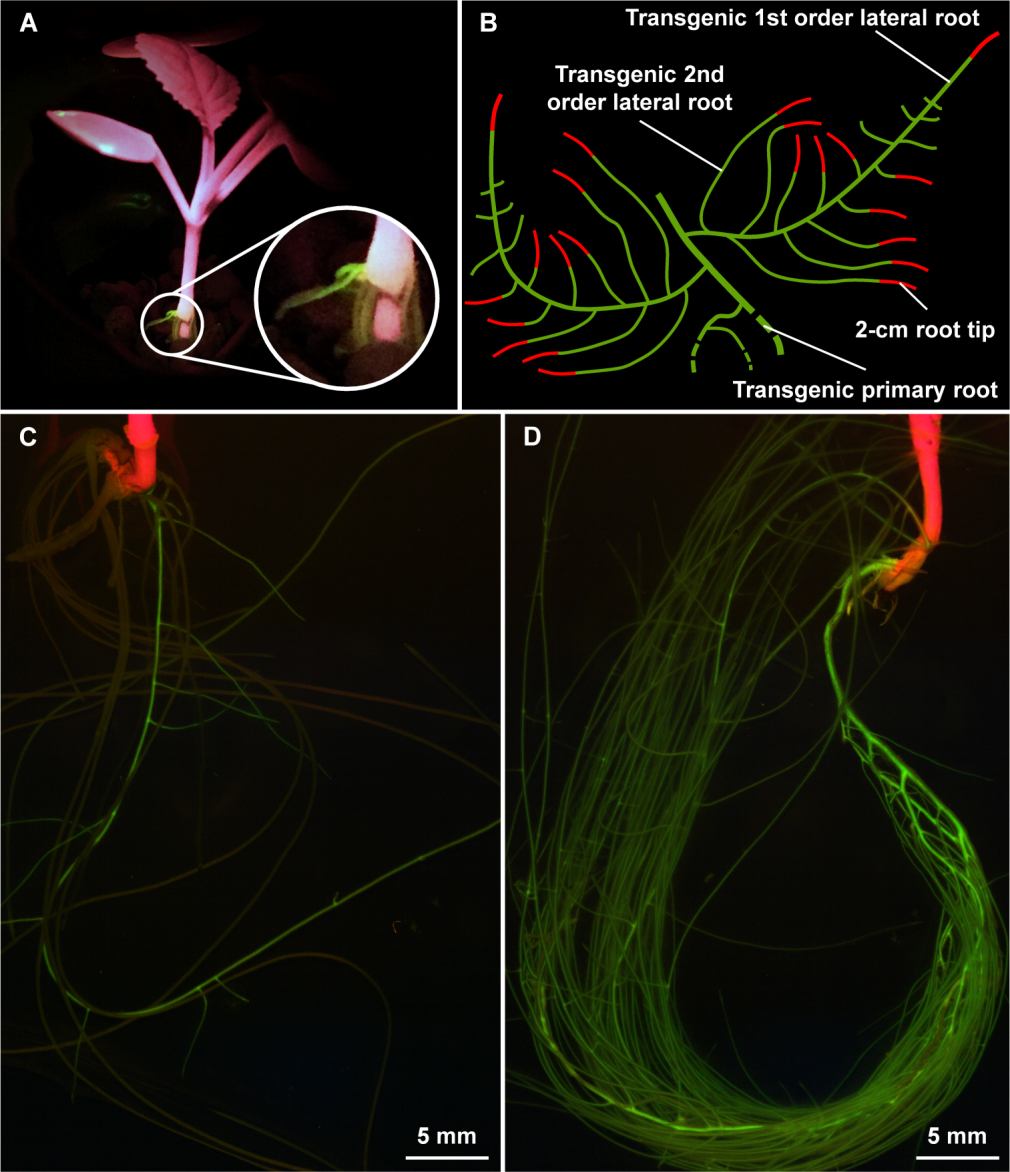
Screening and harvesting of the transgenic root material. Young transgenic root growing out of the inoculation side (A, picture was taken using a blue-green LED flashlight), scheme showing the harvested root material (B), discrimination of transgenic and non-transgenic roots (C) and example of a transgenic root (D) before the harvest (pictures were taken with a fluorescence stereomicroscope).

Upon transformation of the CRISPR construct with the hairy root technique, roots emerging from the inoculation site were transgenic or non-transgenic and a fluorescent marker (GFP) encoded on the vector allowed the selection of transgenic roots by assessing the GFP fluorescence. The transformation efficiency was between two and four percent, whereby one to three transgenic roots were generated per plant. The GFP fluorescence was sufficiently strong to detect a signal with a blue-green LED flashlight within one week, allowing the evaluation of a transformation event early after inoculation without removing the plant from the potting medium (Fig 1A). The GFP signal was equally distributed, showing no indication of gene silencing probably due to the introns included in the GFP coding sequence [29]. Although non-transgenic roots were cut off before the phosphate depletion was started, some of them recovered until the harvest (after eight days of treatment) causing a reduced growth of the transgenic root (Fig 1C) compared to a transgenic root, which was grown alone (Fig 1D). Nevertheless, independent of the growth of the transgenic roots, we harvested a maximum of 2-cm root tips from 1^st^ and 2^nd^ order lateral roots (Fig 1B). In all roots with GFP fluorescence, we were later able to demonstrate evidence for the presence of indels in the genome, suggesting an efficient selection strategy. The position of the GFP gene in the vector close to the left border ensured a proper transfer of the Cas9 gene and the gRNA. As described previously [30], a strong fluorescence signal is putatively correlated with an insertion locus beneficial for transgene expression, suggesting that screening for strong GFP expression may facilitate the selection of roots with a high Cas9 and gRNA expression, resulting in a higher mutation rate.

### Detection of deletions and insertions with PAGE

Initially, we attempted to induce the deletion of a larger fragment within *BcFLA1*, therefore we decided to use a multiplex system, through which it was possible to guide Cas9 to two different sides in the same gene by two gRNAs [23]. As long as the two gRNAs are active at the same time, this will lead to the loss of a large fragment between the two occurring DSBs, so that the knockout of the gene is very likely [31]. However, an initial fragment-analysis by agarose gel electrophoresis suggested that the expected deletion of the fragment between the gRNAs did not occur and Sanger sequencing of DNA amplicons with a size differing from the wildtype allele revealed that the second gRNA induced deletions only very inefficiently (S2 Fig). Nonetheless, although deletions of bigger fragments may ensure the abolishing of gene function, residual fragments resembling the size of the wildtype allele putatively have small indels that cannot be detected by agarose gel electrophoresis and therefore would be misinterpreted as wildtype. Thus, we decided to focus on the detection of small indels rather than producing big deletions, hypothesizing that a precise determination of the amplicon size will reveal a sufficient frequency of the mutational rate for subsequent phenotyping.

**Fig 2:**
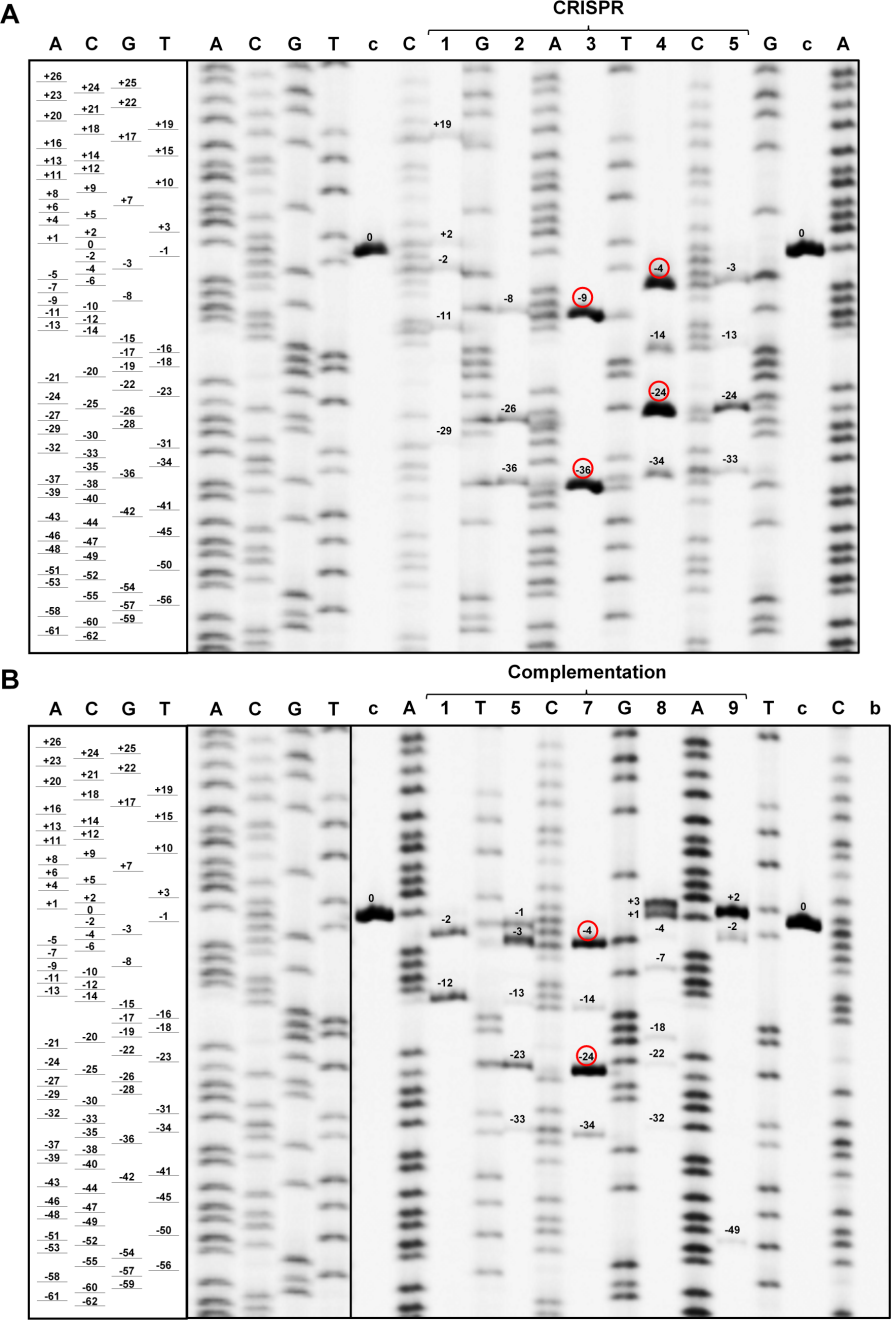
Validation of the CRISPR/Cas9-induced gene editing by polyacrylamide gel electrophoresis. CRISPR samples (A) and complementation samples (B). A, C, G and T = Sanger sequencing reactions with the corresponding dideoxynucleotides; c = control; b = blank (no template control). Lane numbers indicate the respective transgenic roots and are consistent with the following figures. Numbers close to the bands indicate the deleted (-) and inserted (+) bp compared to the wildtype size (0), red circles indicate deletions, which could be validated by Sanger sequencing.

In fact, fragment-analysis by PAGE allowed us to determine the fragment size with a resolution of one base pair (Fig 2) which eliminates the limitation on the size of detectable deletions previously discussed for AFLP [32]. We detected up to 7 fragments (average 4) in one transgenic root and no transgenic roots were found to have an amplicon with the wildtype size. The size of the deletion ranged from 1 to 49 bp and the size of the insertion from 1 to 19 bp. In 26 out of 39 cases a deletion or insertion resulted in a frame shift mutation.

### Validation of deletions and insertions with Sanger sequencing

**Fig 3:**
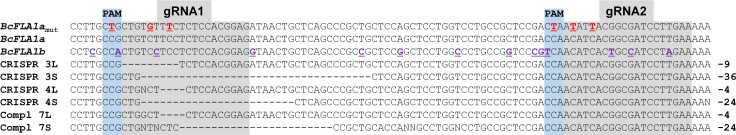
CRISPR/Cas9-induced deletions in *BcFLA1*. Grey background indicates gRNAs, blue background the PAM. Red letters indicate replaced bases in *BcFLA1a*_*mut*_, violet letters differing bases in *BcFLA1b*; numbers on the right indicate deleted bp. There were no deletions around gRNA 2. Compl = complementation. Numbers on the left indicate the respective transgenic roots.

We validated six distinct deletions by sequencing reaching from 4 to 36 bp, whereby two of the samples (CRISPR 4L, and compl 7L) exhibited exactly the same deletion of 4 bp (Fig 3). Most of the sequencing reactions resulted in overlapping of at least two sequences starting at the location of the first gRNA. A completely independent experiment replication revealed twelve further distinct deletions ranging from 3 to 112 bp and three insertions ranging from 6 to 68 bp (S2 Fig). In one case (CRISPR 7S), there were two distinct deletions, one around the first and one around the second gRNA target site. Furthermore, the largest deletion of 112 bp spanned both gRNAs, possibly indicative of two simultaneously active gRNAs. However, all other identified deletions were exclusively around the gRNA1 target site. Additionally, the sequencing revealed that *BcFLA1* had two naturally occurring alleles (a and b). However, although the sequence recognized by the protospacer of gRNA1 was different in one base for *BcFLA1b* (position 15 is A in *BcFLA1a* but G in *BcFLA1b*) (S1C Fig), we detected an efficient induction of deletions and insertions for both alleles (S2 Fig).

The sequence amplified by primers designed to amplify exclusively *BcFLA1a*_*mut*_ matched the mutagenic *BcFLA1* sequence without any deletions.

The separation of the most abundant amplicons of the potentially edited *BcFLA1* gene region, found by fragment analysis by PAGE, was confirmed by Sanger sequencing (Fig 2, Fig 3). However, with fragment analysis by PAGE, we detected weaker bands that could not be analyzed by Sanger sequencing.

### Expression of *BcFLA1* in roots carrying the CRISPR construct

As an independent line of evidence, we monitored the *BcFLA1* expression with qPCR in transgenic roots expressing an inactive (without protospacer) gRNA (control), an active gRNA (CRISPR), and an active gRNA plus *BcFLA1a*_*mut*_ (compl.). We reasoned that indels in *BcFLA1* will result in a frame shift, causing the degradation of the transcript *via* nonsense-mediated mRNA decay (NMD). In the samples complemented with *BcFLA1a*_*mut*_, no deletions and therefore no transcript reduction was expected. To discriminate between the wildtype *BcFLA1* and the *BcFLA1a*_*mut*_ transcript, we tested primer pairs specifically amplifying *BcFLA1* and *BcFLA1a*_*mut*_ (S3 Fig). The *BcFLA1a*_*mut*_-specific primers produced an amplicon only with the complementation plasmid as template, whereas the wildtype-specific primers produced an amplicon exclusively with a wildtype version of *BcFLA1* as a template, confirming primer specificity. Additionally, a third primer pair amplifies the wildtype *BcFLA1* and *BcFLA1a*_*mut*_ equally well providing an estimate of the wildtype *BcFLA1* and *BcFLA1a*_*mut*_ transcript together.

A pretest assessing *BcFLA1* expression using semi-quantitative PCR resulted in a clearly lower expression of the wildtype *BcFLA1* in most of the CRISPR and complementation samples (S4C Fig), which was successfully complemented by the expression of *BcFLA1a*_*mut*_ (S4B and S4D Fig).

**Fig 4:**
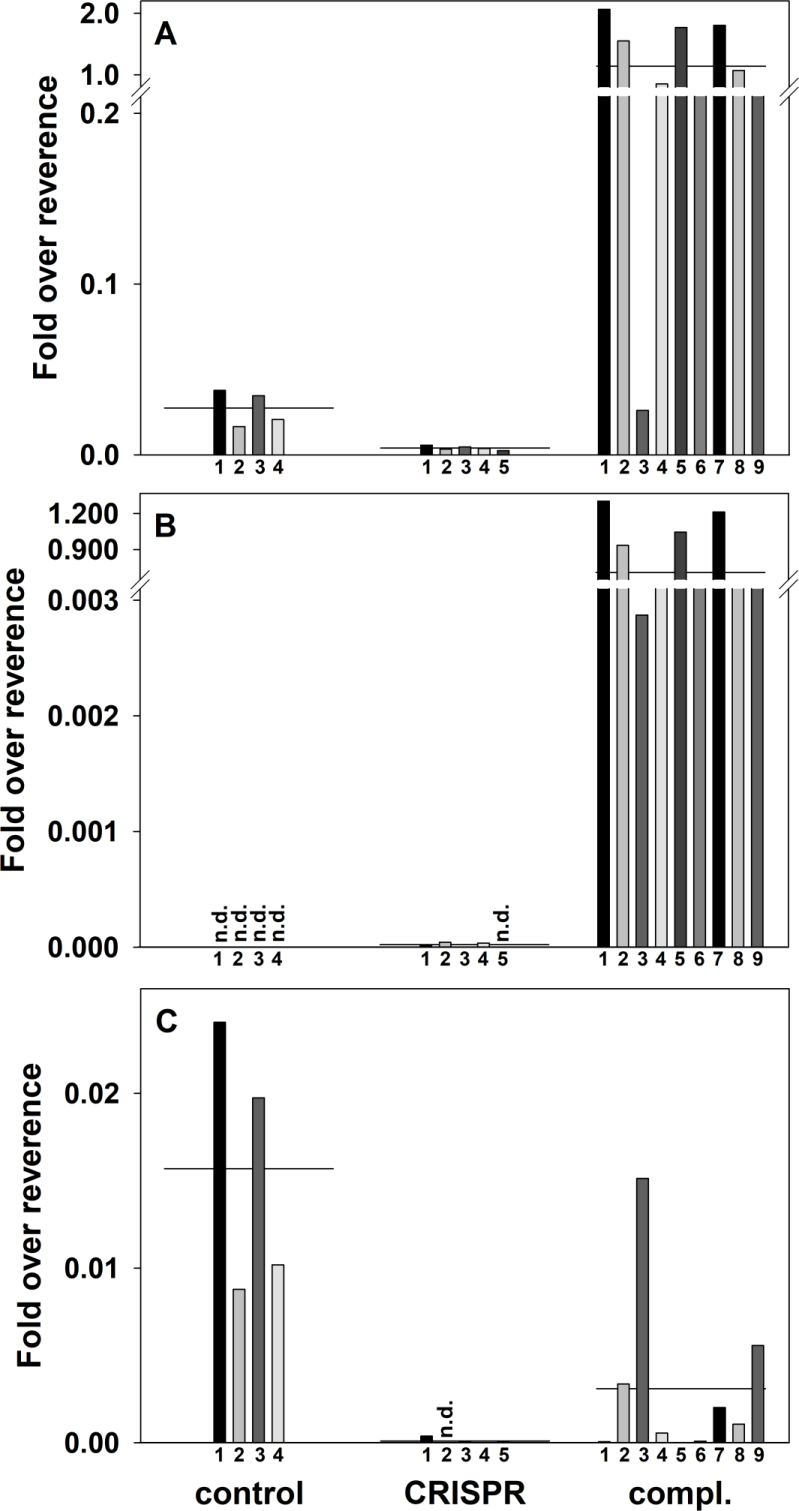
Expression of *BcFLA1* as affected by CRISPR/Cas9-induced gene editing and complementation, respectively. Total *BcFLA1* expression (A), expression of *BcFLA1a*_*mut*_ (B) and expression of the wildtype *BcFLA1* gene (C). Each bar represents the expression in one single transgenic root measured by three technical replicates with the horizontal line representing the corresponding mean of all biological replicates. Numbers indicate the respective transgenic roots. Fold over reverence = 2^-delta Ct^; n.d. = not detected.

A detailed expression analysis by qPCR revealed an average total *BcFLA1* expression of ca. 15% in the CRISPR samples compared to the control (Fig 4A). The same can be observed for wildtype *BcFLA1* where the CRISPR samples had only 0.6% of the transcript compared to the control samples (Fig 4C). This result suggests that a successful induction of deletions and insertions of *BcFLA1* correlates well with the reduction of its transcript.

The complementation of *BcFLA1* by *BcFLA1a*_*mut*_ resulted in more than 40-times higher total *BcFLA1* expression than in the control (Fig 4A). The results show that ectopic expression of *BcFLA1a*_*mut*_ with the ubiquitin promoter (compl.) resulted in much higher transcript abundance than the expression of the wildtype *BcFLA1* with the native promoter (control). This was confirmed with the primer pair specific for *BcFLA1a*_*mut*_, where the average fold over reference was around 0.7 in the complementation samples, which indicated that *BcFLA1a*_*mut*_ was not affected by NMD (Fig 4B). In control and CRISPR samples, the respective transcript was not detectable or negligibly small (< 10^−4^) since *BcFLA1a*_*mut*_ was absent. One out of eight transgenic roots carrying the complementation construct (H280) showed much lower expression of *BcFLA1a*_*mut*_, possibly due to gene silencing effects or a chromosomal position effect regarding the T-DNA insertion site caused by adjacent condensed chromatin [33].

Interestingly, the average expression of the wildtype *BcFLA1* transcript in the complementation samples was about only 20% of that found in the control (compared to 0.6% in the CRISPR samples) (Fig 4C). NMD, causing transcript reduction, seems to be less efficient in the roots carrying the complementation construct compared to the CRISPR construct alone.

An independent biological replication exhibited similar results regarding the expression of *BcFLA1* (S5 Fig).

### Root hair length

**Fig 5:**
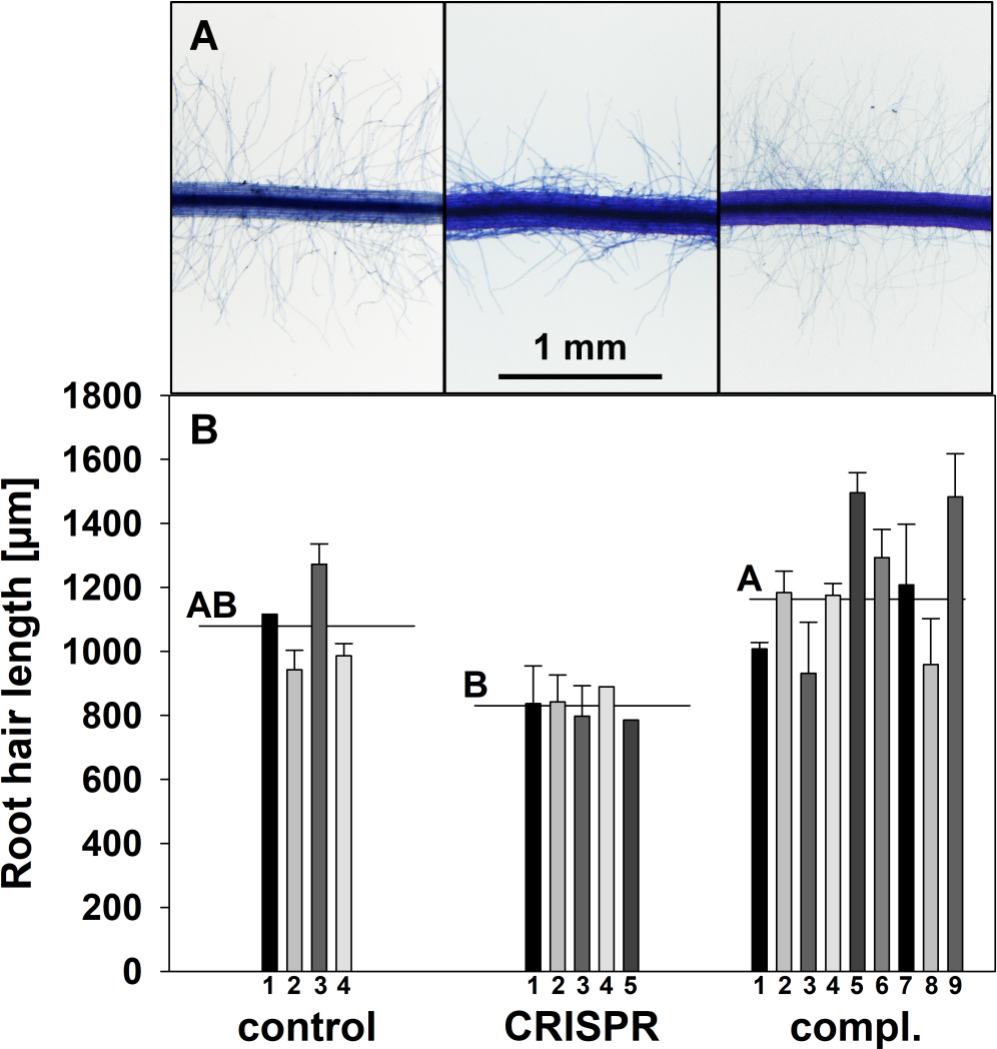
Root hair length of *B*. *carinata* ‘Bale’ as affected by downregulation and complementation of *BcFLA1*, respectively. Root hairs stained by Toluidine Blue (A) and root hair length in μm (B). Each bar represents the mean of the root hair length of root tips from the transgenic 1^st^ and 2^nd^ order lateral root of a single independent event with the error bars indicating the standard error. Numbers indicate the respective transgenic roots. Horizontal lines represent the mean of all transgenic roots. Letters denote significant differences at P < 0.05 (Tukey test).

In the final step, we tested the hypothesis that the deletions and insertions in *BcFLA1* not only affect the open reading frame and transcript expression, but also result in a phenotypic alteration of the root hairs. We scored the root hair length of root tips in the previously characterized transgenic roots and found that compared to the control, transgenic CRISPR roots exhibited a reduced root hair length by trend (Fig 5). In contrast, the complementation of *BcFLA1* led to an increased root hair length compared to the CRISPR roots. The root hairs in the roots carrying the complementation construct were slightly longer than the control roots, which is consistent with the enhanced expression of *BcFLA1* shown by qPCR (Fig 4, S5 Fig).

## Discussion

### An optimized CRISPR construct is required for efficient selection of roots with a disrupted gene function

In this study, we successfully induced site-directed deletions and insertions in the genome of the non-model plant *Brassica carinata* using the CRISPR technology in combination with a hairy root transformation protocol. The efficient generation of these mutations in *BcFLA1* gave us the confidence to directly attempt a phenotypic assessment of the obtained roots. For the success of such a strategy, it is mandatory to optimize the efficiency of the CRISPR construct allowing the medium-throughput selection of individual roots with a disrupted gene function. Primarily, the expression of the Cas9 nuclease, the gRNA, and the fluorescent marker GFP should be under the control of promoters highly active in hairy roots. Hairy roots are most likely chimeras depending on how early the mutation took place. In the case of an early mutation, the expected number of variants in a single transgenic root is smaller. The fact that we found only a few indel events in one individual root (Fig 2) suggests that the proteins needed to induce the mutations were active at an early stage of the transformation so that the mutations, induced in a few founder cells, are carried through the whole root cell population. In conjecture, it was shown previously that the sequence of the protospacer, as well as the location of the target site within the gene, is crucial for the efficiency of the CRISPR construct [14,34]. We designed gRNAs to target a sequence near the 5’ end of *BcFLA1* (S1C Fig) to ensure that the functionally important protein structures, possibly encoded at the beginning of the gene, are affected by the induced deletions. This is consistent with a prior study, in which the 3’ target was less efficient in the gene knockout compared to the 5’ end [14]. A GC content of at least 50% should guarantee a strong binding of the gRNA to the target sequence leading to a higher editing efficiency as observed in a previous study [21]. The latter also determined that pairing of more than 6 bp between the target sequence and the gRNA should be avoided in order to prevent the building of stem-loop structures so that the protospacer is free to bind the target sequence. In a recent publication, the effect of every base at each position of the protospacer was determined for the activity of the gRNA [34]. Interestingly, the higher activity of gRNA1 compared to gRNA2 in our study (only two deletion events were detected for the latter, see Fig 3 and S2 Fig) might be explained by the guanine at position 20 of gRNA1 directly before the PAM sequence as well as the cytosine as variable base of the PAM sequence (S1C Fig), which both are described to result in higher efficiency. In contrast, gRNA2 does not contain these bases at the respective positions and exhibits a thymine as the variable base in the PAM sequence, which was shown to be disfavored [34].

### The relaxed specificity of a gRNA might be compensated by using a complementation construct in parallel

The usage of the CRISPR technology for functional characterization of a gene might be limited by off-target effects affecting additional genes, thereby impeding the association of a mutation with a phenotype. This is especially of concern in a non-model plant without a fully sequenced genome or in cases where the phenotypic consequences of a mutation are evaluated directly without entering the germline, rendering a genetic separation of off-target mutations impossible. In this context, it is noticeable that gRNA1 additionally resulted in gene indel events in another allele of *BcFLA1*, called *BcFLA1b* (S2 Fig). This allele also has a thymine as a variable base in the PAM (S1C Fig), so that gRNA1 possibly had a higher activity for *BcFLA1a* than for *BcFLA1b*. Nevertheless, in *BcFLA1b* the first gRNA was also active even though there was a mismatch at position 15 (6 bp away from the 3’ end) and therefore in the part close to the PAM sequence, which was considered as crucial for the specificity [8,10,11]. However, according to Semenova *et al*. [9], exactly this position is surrounded by, but does not belong to, the so-called ‘seed sequence’, which reflects the base positions in which a perfect base pairing is essential. Moreover, this is consistent with the results of [35], who found that single mismatches, even at the 3’ end of the gRNA, may not lead to a lower efficiency of the gRNA, depending on the target site. We did not systematically screen for off-target effects. However, with the hairy root technique, independent transformation events for each transgenic root were obtained so that the homogeneity of the phenotypic changes indicates that the potential side effects are rather low unless the off-target effects are as frequent as the target effects. Additionally, the impact of such effects on the interpretation of the overall results might be severely reduced by using a complementation approach in parallel. We ectopically expressed *BcFLA1a*_*mut*_ that was resistant to the induction of indels by the Cas9/gRNA combination used for targeting the wildtype *BcFLA1* expressed in parallel (S1C Fig). Both *BcFLA1* genes code for the same amino acid sequence, therefore, the mutant version of *BcFLA1* could complement the loss-of-function caused by indels in the wildtype *BcFLA1*. The success of this approach was monitored by qPCR which was able to distinguish transcripts originating from two different versions of the gene (Fig 4). The expression of *BcFLA1a*_*mut*_ was high in transgenic roots carrying the complementation construct and therefore seemed unaffected by the Cas9/gRNA designed to target wildtype *BcFLA1* (Fig 4B). However, the reduction of the wildtype *BcFLA1* expression in the complementation samples was not as clear as in the CRISPR samples (Fig 4C). This may be due to a reduced efficiency of the CRISPR/Cas9 mechanism in the complementation vector. It is conceivable that the strong overexpression of *BcFLA1a*_*mut*_ leads to a reduced expression of the Cas9 protein because the expression of both is under the control of the same ubiquitin promoter from parsley. Another reason may be that the wildtype primer also binds to *BcFLA1a*_*mut*_ with a very low rate, which was not detectable in the agarose gel (S3 Fig), but measurable by the more sensitive qPCR. Even if this rate is very low, it may have an influence because of the extremely high expression of the ectopically expressed *BcFLA1a*_*mut*_. Another possibility is a feedback regulation of the overexpressed *BcFLA1a*_*mut*_ on the wildtype *BcFLA1* expression, suggesting that the expression of *BcFLA1a*_*mut*_ under control of the native promoter might be a significant improvement of the system.

### The size of deletions caused by CRISPR in hairy roots is highly variable

In this study, we found deletions ranging from 1 to 112 bp, some of which were easily detectable in a fragment analysis by agarose gel electrophoresis. Indeed, it was possible to identify deletions and sometimes even insertions for many of the samples (Fig 3, S2 Fig) with this approach. Even though there are examples of larger deletions [36,37], CRISPR/Cas9 studies applied in systems other than hairy roots, usually led to deletions of only a few bases as long as only one gRNA was used [6,7]. Perhaps, the hairy root transformation leads to larger deletions than other transformation methods, for example, because the DSB repair mechanisms in hairy roots may be less efficient than in other plant tissues or the activity of nucleases degrading the DNA strands before the repair is higher. However, in another paper working with CRISPR/Cas9 in hairy roots, mostly the usual 1 or 2 bp indels and only one 5 bp and one 7 bp deletion were observed at two different sites targeted by one gRNA each [17]. Furthermore, Michno *et al*. [16] detected, with one exception of 32 bp, only small (up to 8 bp) CRISPR/Cas9-induced deletions in hairy roots in overall three different target sites and two different plant species. Hence, it is also possible that the target site is decisive for the type and size of the observed gene editing events, as expected by Jacobs *et al*. [14]. The latter also applied CRISPR/Cas9 in a hairy root system and observed on average smaller deletions than in our study, but depending on the target sites, large indels were also detected. Furthermore, the authors observed similar mutations for the same target sites in somatic embryos, independent of the transformation method. Wang *et al*. [18] also applied CRISPR/Cas9 in hairy roots and observed 1–2 bp indels induced by a single gRNA, and larger deletions using two gRNAs. Interestingly, deletions larger than the sequence between the two gRNAs were detected. Summing up, the type and size of indels differs strongly depending on the target site and sometimes also between hairy roots of the same target and even within a single hairy root. The latter was also observed by Iaffaldano *et al*. [15], who detected for the same target in one hairy root culture exclusively 1 bp indels and in three others varying indels between 1 and up to 22 bp.

### Sensitive detection of indels with fragment analysis *via* PAGE

For the detection of small indels by fragment analysis the resolution of agarose gels is too low, therefore we tried to identify a method that is sensitive, semi-quantitative, cost-effective and able to estimate the size of the deletions. Jacobs *et al*. [14] used next generation sequencing to detect and quantify all mutations that were induced by CRISPR/Cas9 in hairy roots. This is a highly effective, though a costly approach. Additionally, cleaved amplified polymorphic sequence (CAPS) assays were used to detect the mutations [15–18]. However, this method requires a restriction site in the gRNA target sequence which is destroyed by a deletion in the mutant. This limits the number of suitable target sequences so that the design of specific and efficient gRNAs is more difficult. Another method, the high-resolution melting curve analysis, is effective and sensitive in detecting mutations [38], but cannot determine the exact number and size of indels. We decided to combine an amplicon size analysis strategy with the high resolution of PAGE. Indeed, the size of the deletions was determined with one base pair resolution and deletions were revealed in all samples (Fig 2). In all cases, there was no signal indicative of the wildtype allele without deletions detectable. The results from the fragment analysis by PAGE were in agreement with the Sanger sequencing obtained previously from fragments isolated from agarose gel electrophoresis (Fig 3). Additionally, the reduction of *BcFLA1* transcript measured by qPCR is an independent line of evidence that suggests a successful induction of site-directed indels (Fig 4). However, this method cannot be used to quantify the effect. We believe that fragment analysis by PAGE is uniquely suited for the detection of indels, especially when the chance of occurrence of a wildtype allele in the sample should be minimized. However, a direct evaluation of the fragment analysis by PAGE by next-generation sequencing might help to further establish the sensitivity and the accuracy of the method. Furthermore, we would like to emphasize that the amount of DNA loaded is crucial for sensitivity and accuracy of the fragment analysis by PAGE. In fact, overloading of the gel ensures that there are indeed no additional amplicons at a low level. However, this impedes a good estimation of the deletion size. Furthermore, the resolution of the PAGE is substantially increased by use of amplicons smaller than 250 bp.

### Effect of indels in *BcFLA1* on root hair length

Because of the proposed role of *BcFLA1* in the root hair elongation, we expected an influence of its loss-of-function on the root hair length. Indeed, the root hairs of the transgenic CRISPR roots were by tendency shorter compared to that of the wildtype (Fig 5). In order to exclude off-target effects, we additionally complemented the gene function of *BcFLA1* by simultaneously overexpressing a mutant *BcFLA1a*_*mut*_ gene, which was immune against the CRISPR/Cas9 mechanism. The consequence was not only the recovery of the wildtype root hair length but even the formation of slightly longer root hairs (Fig 5). This fits with the expression of *BcFLA1* in the complementation samples, which was on average 40-times higher than in the wildtype (Fig 4A). In consideration of the proposed role of *BcFLA1* in the root hair formation, the effect of the complementation of *BcFLA1* and the fact that off-target effects in plants are less frequent [6,7,14], the effect on the root hair length can be traced back to a successful disruption of *BcFLA1* gene function by CRISPR/Cas9. We believe the direct phenotyping of roots harboring deletions originating from CRISPR/Cas9, in combination with the hairy root technique, is a versatile tool, especially when the root is a focus of the research. We envision a great impact on studies e.g. in plant nutrition, plant pathogen, and plant symbiont interaction as well as root developmental biology.

## Conclusion

The objective of this paper was the application of the CRISPR/Cas9 system by performing a stable transformation of *Brassica carinata* using the hairy root technique. Gene editing could be verified indirectly by qPCR as well as directly by Sanger sequencing and fragment analysis by PAGE. Additionally, we could demonstrate a phenotypic effect of the gene editing on the root hair length. Fragment analysis by PAGE turned out to be an especially successful method to uncover all gene editing events.

## Supporting information

S1 Fig Expression cassette and *BcFLA1* gRNAs. Expression cassette of CRISPR+35S::GFP+fla1-guides (A), the complementation vector (B) and gRNAs for CRISPR/Cas9 targeting *BcFLA1* (C). For the control, the pB-CRISPR+35S::GFP vector without gRNAs was introduced into the plants. The gRNA sequences are displayed in the reverse and complement form for a better understanding.
(TIF)

S2 Fig CRISPR/Cas9-induced deletions and insertions in *BcFLA1*. Grey background indicates the region of the gRNAs; violet letters indicate differing bases in *BcFLA1b*, from which the first two were used two discriminate between both alleles; inserted bp (red numbers) and deleted bp (on the right). Data from the independent experiment replication. Compl = complementation. Numbers on the left indicate the respective transgenic roots.
(TIF)

S3 Fig *BcFLA1* primers are specific for the mutant and the wildtype version of the gene, respectively. Primers for the validation of the gene editing with large products (A) and small products (B). Primers used for expression analysis (C). C = control sample, P = plasmid used for the complementation, N = no template control.
(TIF)

S4 Fig Pretest of the qPCR primers confirming the primer specificity and revealing a successful downregulation and complementation. Expression of the endogenous control *AtUBC9* (A), *BcFLA1a*_*mut*_ (B), the wildtype *BcFLA1* (C) and both versions of *BcFLA1* (D). Data from independently replicated experiment.
(TIF)

S5 Fig Expression of *BcFLA1* as affected by CRISPR/Cas9-induced gene editing and complementation, respectively. Total *BcFLA1* expression (A), expression of *BcFLA1a*_*mut*_ (B) and expression of the wildtype *BcFLA1* gene (C). Each bar represents the expression in one single transgenic root measured by three technical replicates with the horizontal line representing the corresponding mean of all biological replicates. Data from independently replicated experiment. Numbers indicate the respective transgenic roots. Fold over reverence = 2^-delta Ct^; n.d. = not detected.
(TIF)

S1 Text Preparation of the constructs. (DOCX)

S2 Text Expression analysis. (DOCX)

S1 Table Primers used for construct preparation. (DOCX)

S2 Table qPCR primers. Wildtype and mutant-specific primers were designed on the two gRNA regions harboring three differing bases at the 3’ end (underlined). The unspecific primers are located at the 3’ end of *BcFLA1*.
(DOCX)

S3 Table Primer pairs for the verification of gene editing. For M13 SSR-PCR forward primers were tagged with M13 tail GTAAAACGACGGCCAGT. Numbers in brackets indicate amplicon size including the M13 tail.
(DOCX)

## References

[1] Shan G. RNA interference as a gene knockdown technique. Int J Biochem Cell Biol. 2010; 42: 1243–1251. doi: 10.1016/j.biocel.2009.04.023 19442757 10.1016/j.biocel.2009.04.023

[2] Belhaj K, Chaparro-Garcia A, Kamoun S, Patron NJ, Nekrasov V. Editing plant genomes with CRISPR/Cas9. Curr Opin Biotechnol. 2015; 32: 76–84. doi: 10.1016/j.copbio.2014.11.007 25437637 10.1016/j.copbio.2014.11.007

[3] Zhang D, Li Z, Li J-F. Targeted gene manipulation in plants using the CRISPR/Cas technology. J Genet Genomics. 2016; 43: 251–262. doi: 10.1016/j.jgg.2016.03.001 27165865 10.1016/j.jgg.2016.03.001

[4] Barrangou R, Birmingham A, Wiemann S, Beijersbergen RL, Hornung V, van Smith AB. Advances in CRISPR-Cas9 genome engineering. Lessons learned from RNA interference. Nucleic Acids Res. 2015; 43: 3407–3419. doi: 10.1093/nar/gkv226 25800748 10.1093/nar/gkv226 PMC4402539

[5] Knoll A, Fauser F, Puchta H. DNA recombination in somatic plant cells. Mechanisms and evolutionary consequences. Chromosome Res. 2014; 22: 191–201. doi: 10.1007/s10577-014-9415-y 24788060 10.1007/s10577-014-9415-y

[6] Feng Z, Mao Y, Xu N, Zhang B, Wei P, Yang D-L, et al Multigeneration analysis reveals the inheritance, specificity, and patterns of CRISPR/Cas-induced gene modifications in Arabidopsis. Proc Natl Acad Sci U S A. 2014; 111: 4632–4637. doi: 10.1073/pnas.1400822111 24550464 10.1073/pnas.1400822111 PMC3970504

[7] Zhang H, Zhang J, Wei P, Zhang B, Gou F, Feng Z, et al The CRISPR/Cas9 system produces specific and homozygous targeted gene editing in rice in one generation. Plant Biotechnol J. 2014; 12: 797–807. doi: 10.1111/pbi.12200 24854982 10.1111/pbi.12200

[8] Cong L, Ran FA, Cox D, Lin S, Barretto R, Habib N, et al Multiplex genome engineering using CRISPR/Cas systems. Science. 2013; 339: 819–823. doi: 10.1126/science.1231143 23287718 10.1126/science.1231143 PMC3795411

[9] Semenova E, Jore MM, Datsenko KA, Semenova A, Westra ER, Wanner B, et al Interference by clustered regularly interspaced short palindromic repeat (CRISPR) RNA is governed by a seed sequence. Proc Natl Acad Sci U S A. 2011; 108: 10098–10103. doi: 10.1073/pnas.1104144108 21646539 10.1073/pnas.1104144108 PMC3121866

[10] Jinek M, Chylinski K, Fonfara I, Hauer M, Doudna JA, Charpentier E. A programmable dual-RNA-guided DNA endonuclease in adaptive bacterial immunity. Science. 2012; 337: 816–821. doi: 10.1126/science.1225829 22745249 10.1126/science.1225829 PMC6286148

[11] Jiang W, Bikard D, Cox D, Zhang F, Marraffini LA. RNA-guided editing of bacterial genomes using CRISPR-Cas systems. Nat Biotechnol. 2013; 31: 233–239. doi: 10.1038/nbt.2508 23360965 10.1038/nbt.2508 PMC3748948

[12] Bortesi L, Fischer R. The CRISPR/Cas9 system for plant genome editing and beyond. Biotechnol Adv. 2015; 33: 41–52. doi: 10.1016/j.biotechadv.2014.12.006 25536441 10.1016/j.biotechadv.2014.12.006

[13] Ding Y, Li H, Chen L-L, Xie K. Recent advances in genome editing using CRISPR/Cas9. Front Plant Sci. 2016; 7: 703 doi: 10.3389/fpls.2016.00703 27252719 10.3389/fpls.2016.00703 PMC4877526

[14] Jacobs TB, LaFayette PR, Schmitz RJ, Parrott WA. Targeted genome modifications in soybean with CRISPR/Cas9. BMC Biotechnol. 2015; 15: 16 doi: 10.1186/s12896-015-0131-2 25879861 10.1186/s12896-015-0131-2 PMC4365529

[15] Iaffaldano B, Zhang Y, Cornish K. CRISPR/Cas9 genome editing of rubber producing dandelion *Taraxacum kok-saghyz* using *Agrobacterium rhizogenes* without selection. Ind Crops Prod. 2016; 89: 356–362. doi: 10.1016/j.indcrop.2016.05.029

[16] Michno J-M, Wang X, Liu J, Curtin SJ, Kono TJ, Stupar RM. CRISPR/Cas mutagenesis of soybean and *Medicago truncatula* using a new web-tool and a modified Cas9 enzyme. GM Crops Food. 2015; 6: 243–252. doi: 10.1080/21645698.2015.1106063 26479970 10.1080/21645698.2015.1106063 PMC5033229

[17] Ron M, Kajala K, Pauluzzi G, Wang D, Reynoso MA, Zumstein K, et al Hairy root transformation using *Agrobacterium rhizogenes* as a tool for exploring cell type-specific gene expression and function using tomato as a model. Plant Physiol. 2014; 166: 455–469. doi: 10.1104/pp.114.239392 24868032 10.1104/pp.114.239392 PMC4213079

[18] Wang L, Wang L, Tan Q, Fan Q, Zhu H, Hong Z, et al Efficient inactivation of symbiotic nitrogen fixation related genes in *Lotus japonicus* using CRISPR-Cas9. Front Plant Sci. 2016; 7: 1333 doi: 10.3389/fpls.2016.01333 27630657 10.3389/fpls.2016.01333 PMC5006320

[19] Veena V, Taylor CG. *Agrobacterium rhizogenes*. Recent developments and promising applications. In Vitro Cell Dev Biol Plant. 2007; 43: 383–403. doi: 10.1007/s11627-007-9096-8

[20] Kahl G, Molina C, Rotter B, Jüngling R, Frank A, Krezdorn N, et al Reduced representation sequencing of plant stress transcriptomes. J Plant Biochem Biotechnol. 2012; 21: 119–127. doi: 10.1007/s13562-012-0129-y

[21] Ma X, Zhang Q, Zhu Q, Liu W, Chen Y, Qiu R, et al A robust CRISPR/Cas9 system for convenient, high-efficiency multiplex genome editing in monocot and dicot plants. Mol Plant. 2015; 8: 1274–1284. doi: 10.1016/j.molp.2015.04.007 25917172 10.1016/j.molp.2015.04.007

[22] Zuker M. Mfold web server for nucleic acid folding and hybridization prediction. Nucleic Acids Res. 2003; 31: 3406–3415. doi: 10.1093/nar/gkg595 12824337 10.1093/nar/gkg595 PMC169194

[23] Xie K, Minkenberg B, Yang Y. Boosting CRISPR/Cas9 multiplex editing capability with the endogenous tRNA-processing system. Proc Natl Acad Sci U S A. 2015; 112: 3570–3575. doi: 10.1073/pnas.1420294112 25733849 10.1073/pnas.1420294112 PMC4371917

[24] Vieweg MF, Frühling M, Quandt H-J, Heim U, Bäumlein H, Pühler A, et al The promoter of the *Vicia faba* L. leghemoglobin gene VfLb29 is specifically activated in the infected cells of root nodules and in the arbuscule-containing cells of mycorrhizal roots from different legume and nonlegume plants. Mol Plant Microbe Interact. 2004; 17: 62–69. doi: 10.1094/MPMI.2004.17.1.62 14714869 10.1094/MPMI.2004.17.1.62

[25] Vandesompele J, Preter K de, Pattyn F, Poppe B, van Roy N, Paepe A de, et al Accurate normalization of real-time quantitative RT-PCR data by geometric averaging of multiple internal control genes. Genome Biol. 2002; 3: 1–12. doi: 10.1186/gb-2002-3-7-research0034 10.1186/gb-2002-3-7-research0034 PMC126239 12184808

[26] Untergasser A, Cutcutache I, Koressaar T, Ye J, Faircloth BC, Remm M, et al Primer3—new capabilities and interfaces. Nucleic Acids Res. 2012; 40: e115 doi: 10.1093/nar/gks596 22730293 10.1093/nar/gks596 PMC3424584

[27] Schuelke M. An economic method for the fluorescent labeling of PCR fragments. Nat Biotechnol. 2000; 18: 233–234. doi: 10.1038/72708 10657137 10.1038/72708

[28] Seifert GJ, Roberts K. The biology of arabinogalactan proteins. Annu Rev Plant Biol. 2007; 58: 137–161. doi: 10.1146/annurev.arplant.58.032806.103801 17201686 10.1146/annurev.arplant.58.032806.103801

[29] Dadami E, Moser M, Zwiebel M, Krczal G, Wassenegger M, Dalakouras A. An endogene-resembling transgene delays the onset of silencing and limits siRNA accumulation. FEBS Lett. 2013; 587: 706–710. doi: 10.1016/j.febslet.2013.01.045 23380068 10.1016/j.febslet.2013.01.045

[30] Harper BK, Mabon SA, Leffel SM, Halfhill MD, Richards HA, Moyer KA, et al Green fluorescent protein as a marker for expression of a second gene in transgenic plants. Nat Biotechnol. 1999; 17: 1125–1129. doi: 10.1038/15114 10545923 10.1038/15114

[31] Zhou H, Liu B, Weeks DP, Spalding MH, Yang B. Large chromosomal deletions and heritable small genetic changes induced by CRISPR/Cas9 in rice. Nucleic Acids Res. 2014; 42: 10903–10914. doi: 10.1093/nar/gku806 25200087 10.1093/nar/gku806 PMC4176183

[32] Zischewski J, Fischer R, Bortesi L. Detection of on-target and off-target mutations generated by CRISPR/Cas9 and other sequence-specific nucleases. Biotechnol Adv. 2017; 35: 95–104. doi: 10.1016/j.biotechadv.2016.12.003 28011075 10.1016/j.biotechadv.2016.12.003

[33] West AG, Gaszner M, Felsenfeld G. Insulators. Many functions, many mechanisms. Gene Dev. 2002; 16: 271–288. doi: 10.1101/gad.954702 11825869 10.1101/gad.954702

[34] Doench JG, Hartenian E, Graham DB, Tothova Z, Hegde M, Smith I, et al Rational design of highly active sgRNAs for CRISPR-Cas9-mediated gene inactivation. Nat Biotechnol. 2014; 32: 1262–1267. doi: 10.1038/nbt.3026 25184501 10.1038/nbt.3026 PMC4262738

[35] Fu Y, Foden JA, Khayter C, Maeder ML, Reyon D, Joung JK, et al High-frequency off-target mutagenesis induced by CRISPR-Cas nucleases in human cells. Nat Biotechnol. 2013; 31: 822–826. doi: 10.1038/nbt.2623 23792628 10.1038/nbt.2623 PMC3773023

[36] Lawrenson T, Shorinola O, Stacey N, Li C, Ostergaard L, Patron N, et al Induction of targeted, heritable mutations in barley and *Brassica oleracea* using RNA-guided Cas9 nuclease. Genome Biol. 2015; 16: 258 doi: 10.1186/s13059-015-0826-7 26616834 10.1186/s13059-015-0826-7 PMC4663725

[37] Fauser F, Schiml S, Puchta H. Both CRISPR/Cas-based nucleases and nickases can be used efficiently for genome engineering in *Arabidopsis thaliana*. Plant J. 2014; 79: 348–359. doi: 10.1111/tpj.12554 24836556 10.1111/tpj.12554

[38] Wang K, Mei DY, Liu QN, Qiao XH, Ruan WM, Huang T, et al Research of methods to detect genomic mutations induced by CRISPR/Cas systems. J Biotechnol. 2015; 214: 128–132. doi: 10.1016/j.jbiotec.2015.09.029 26419205 10.1016/j.jbiotec.2015.09.029

