# Supplementary figures and images for: Efficient generation of mutations mediated by CRISPR/Cas9 in the hairy root transformation system of Brassica carinata
Source: PLoS One. 2017 Sep 22;12(9):e0185429. doi: 10.1371/journal.pone.0185429 (PMC5609758; doi:10.1371/journal.pone.0185429)

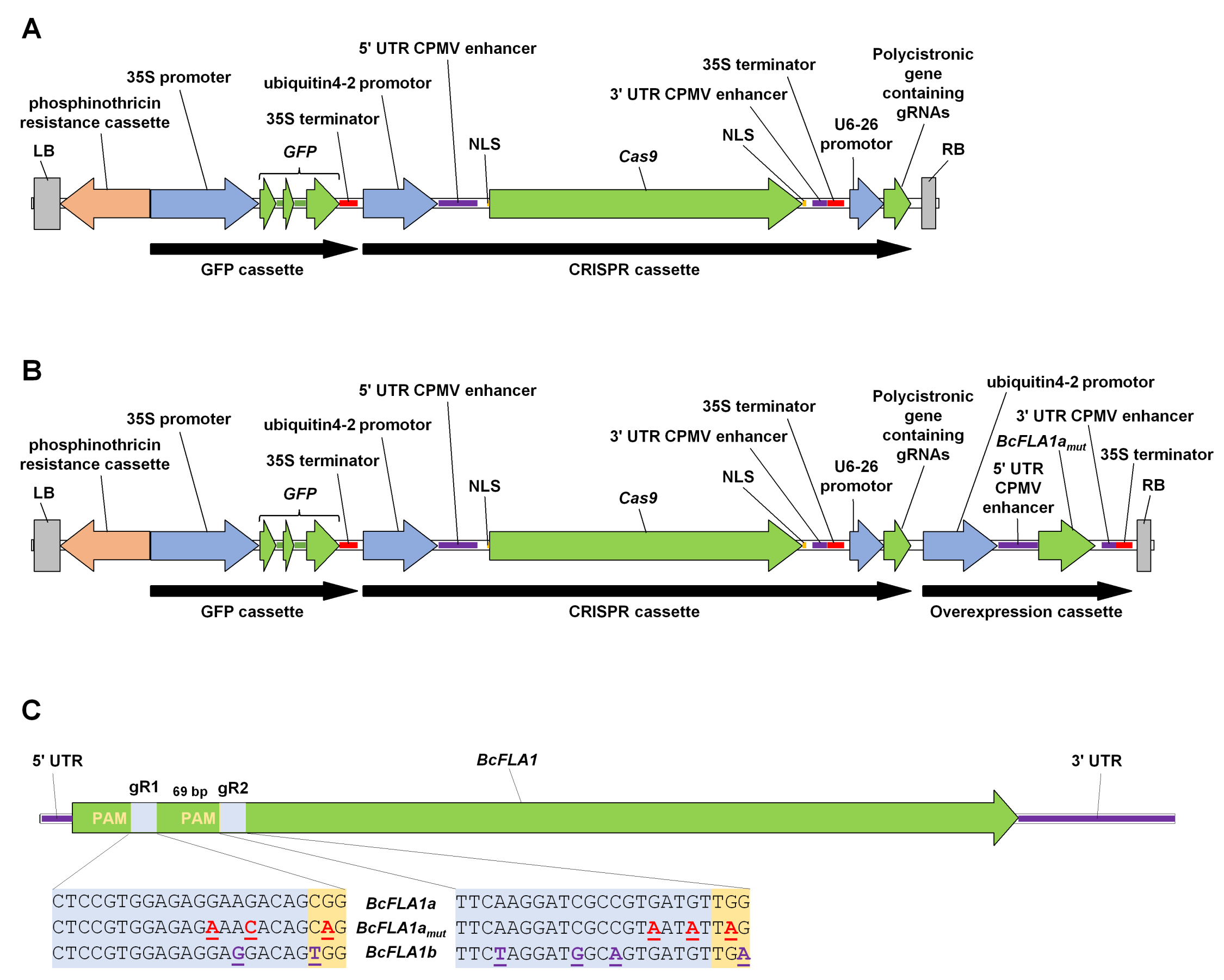

Supplement: S1 Fig — Expression cassette of CRISPR+35S::GFP+fla1-guides (A), the complementation vector (B) and gRNAs for CRISPR/Cas9 targeting BcFLA1 (C). For the control, the pB-CRISPR+35S::GFP vector without gRNAs was introduced into the plants. The gRNA sequences are displayed in the reverse and complement form for a better understanding. (TIF) [file pone.0185429.s001.tif]

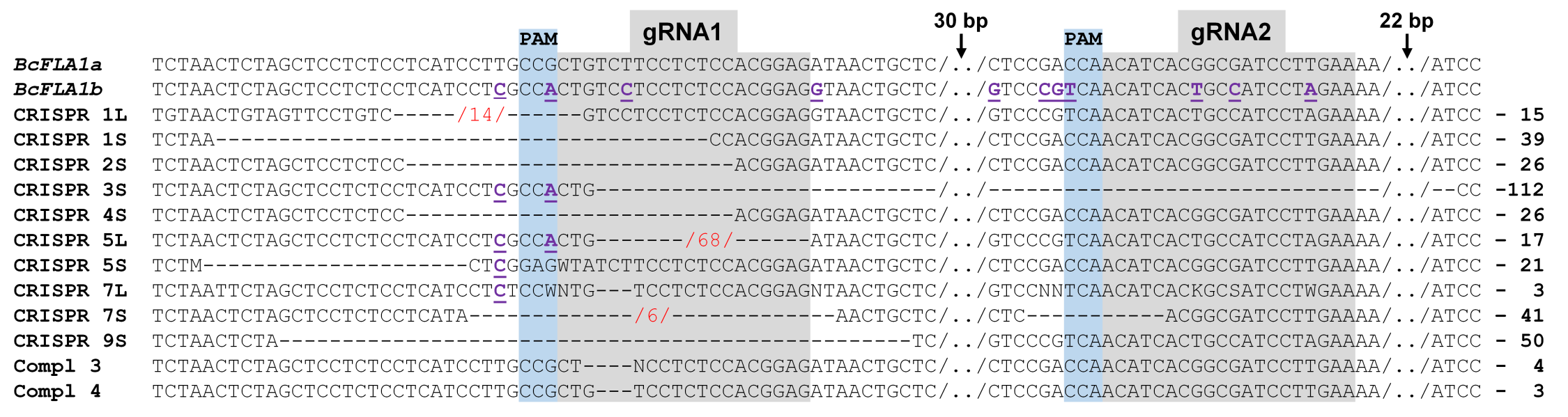

Supplement: S2 Fig — Grey background indicates the region of the gRNAs; violet letters indicate differing bases in BcFLA1b, from which the first two were used two discriminate between both alleles; inserted bp (red numbers) and deleted bp (on the right). Data from the independent experiment replication. Compl = complementation. Numbers on the left indicate the respective transgenic roots. (TIF) [file pone.0185429.s002.tif]

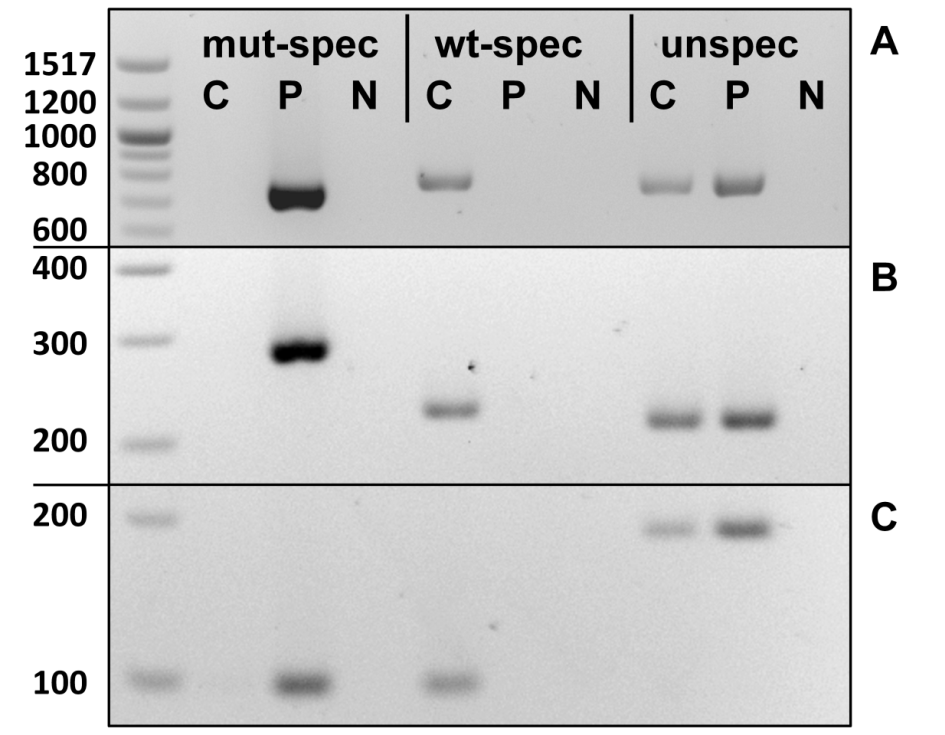

Supplement: S3 Fig — Primers for the validation of the gene editing with large products (A) and small products (B). Primers used for expression analysis (C). C = control sample, P = plasmid used for the complementation, N = no template control. (TIF) [file pone.0185429.s003.tif]

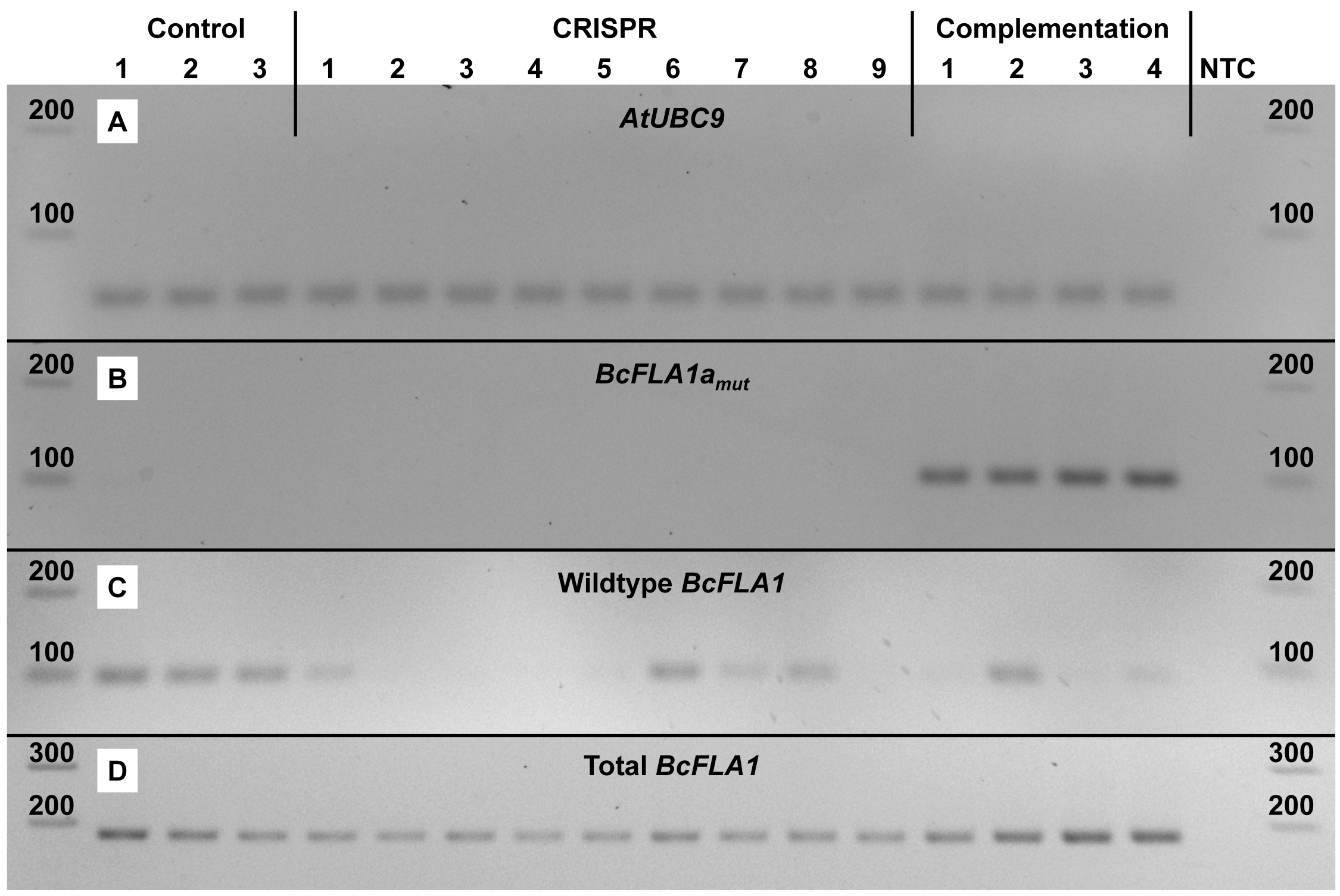

Supplement: S4 Fig — Expression of the endogenous control AtUBC9 (A), BcFLA1amut (B), the wildtype BcFLA1 (C) and both versions of BcFLA1 (D). Data from independently replicated experiment. (TIF) [file pone.0185429.s004.tif]

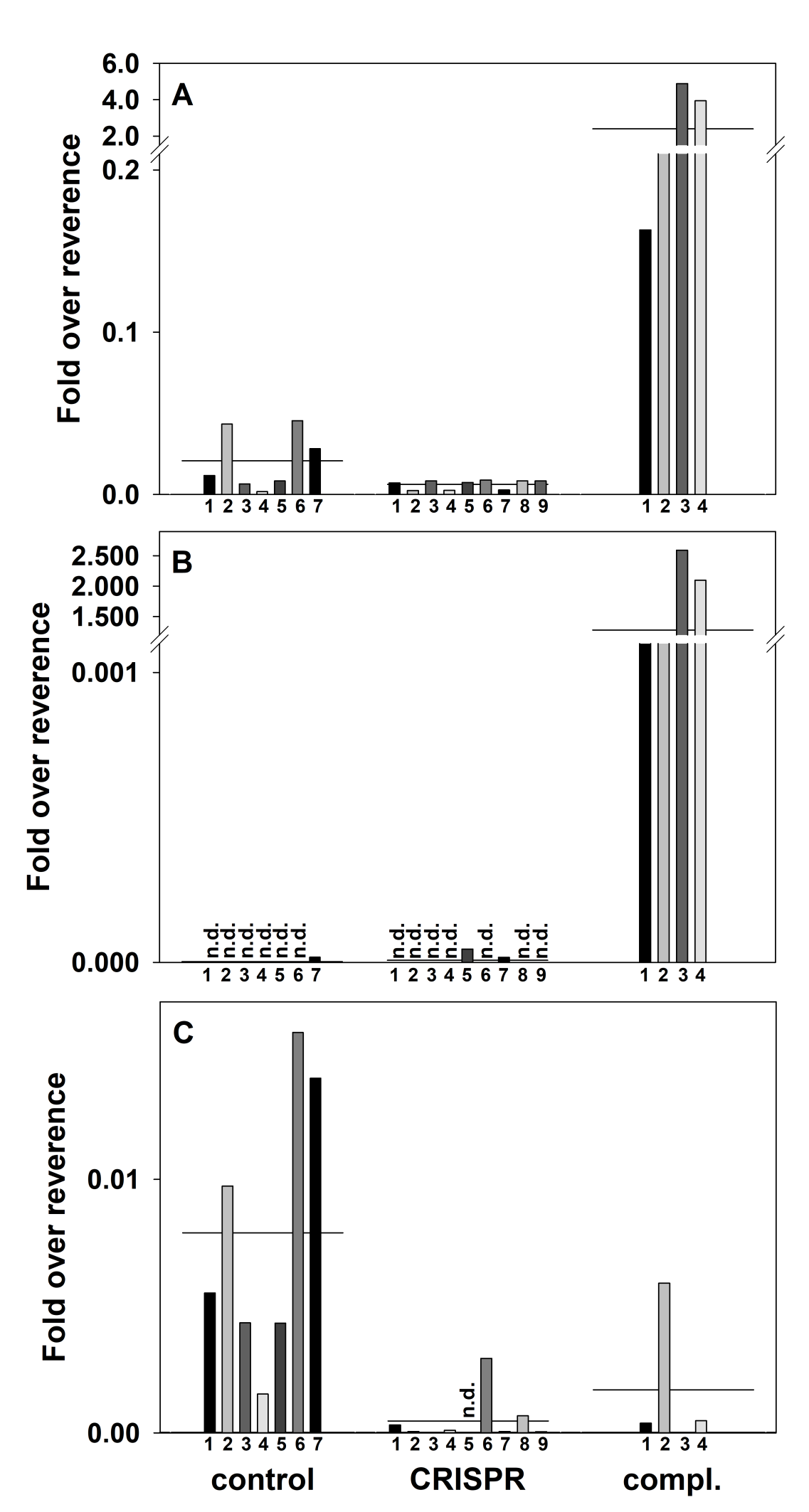

Supplement: S5 Fig — Total BcFLA1 expression (A), expression of BcFLA1amut (B) and expression of the wildtype BcFLA1 gene (C). Each bar represents the expression in one single transgenic root measured by three technical replicates with the horizontal line representing the corresponding mean of all biological replicates. Data from independently replicated experiment. Numbers indicate the respective transgenic roots. Fold over reverence = 2-delta Ct; n.d. = not detected. (TIF) [file pone.0185429.s005.tif]
