# Supplementary material for: Efficient generation of mutations mediated by CRISPR/Cas9 in the hairy root transformation system of Brassica carinata
Source: PLoS One. 2017 Sep 22;12(9):e0185429. doi: 10.1371/journal.pone.0185429 (PMC5609758; doi:10.1371/journal.pone.0185429)
Supplement: S1 Text — (DOCX) [file pone.0185429.s006.docx]

**S1 Text.** **Cloning of the constructs.**

Primer Ubi-sense and Ubi-anti (S1 Table) were used to amplify the ubiquitin4-2 promoter from parsley using plasmid pPZP221-I-SceI_co as a template (kind gift of the Puchta lab in Karlsruhe, see Fauser *et al*. (2012)) with attached AscI and XhoI restriction sites and cloned into the AscI/XhoI restricted V69 vector (containing modified 5’ and 3’ UTRs of RNA-2 from the Cowpea Mosaic Virus as translational enhancers, see Myrach *et al*. (2017) for a detailed description of V69) replacing the 35S promoter with the ubiquitin4-2 promoter resulting in V69-Ubi. The primers Cas9-sense and Cas9-anti were used to amplify the Cas9 gene from 35S-Cas9-SK (kindly provided by Jian-Kang Zhu, Shanghai Center for Plant Stress Biology, China, see Feng *et al*. (2013) and Mao *et al*. (2013)) with attached EcoRI/XmaI sites and the Cas9 gene was cloned into EcoRI and XmaI linearized V69-Ubi resulting in V69-Ubi:Cas9. With hybridized oligos MCS1 and MCS2 a multiple cloning site with MluI, KpnI, AgeI and AatII sites was added to the PmeI and NotI linearized V69-Ubi:Cas9 resulting in V69-Ubi-Cas9-MCS. The U6-26 promoter together with the guide scaffold (separated by a BbsI cloning site allowing the cloning of the target sequence) was amplified from the vector AtU6-26-SK (also kindly provided by Jian-Kang Zhu) with primer guide-RNA-sense and guide-RNA-anti adding flanking KpnI and MluI sites and the KpnI/MluI restricted PCR product was ligated in the KpnI/MluI linearized V69-Ubi:Cas9-MCS resulting in V69-Ubi:Cas9-MCS-U6. For amplification of a fluorescent marker with introns for reduced silencing we used pG104-GFPendo (kindly provided by Michael Wassenegger, see Dadami *et al*. (2013) for details) as a template. The gene cassette including rbcS promoter and terminator contained BbsI sites that were removed by amplification of four overlapping PCR-products (GFP-1+GFP-2, GFP-3+GFP-4, GFP-5+GFP-6, GFP-7+GFP8) that were assembled together with the AscI linearized V69-Ubi:Cas9-MCS-U6 vector using the NEBuilder HiFi DNA Assembly Cloning Kit (New England Biolabs, Ipswich, USA) creating the vector V69-Ubi-Cas9-MCS-U6-rbcS:GFP (V110). The backbone of the binary vector was switched by cloning the complete cassette into pBGWFS (BbsI sites were removed previously) using PmeI and Eco47III, resulting in pB-Ubi-Cas9-MCS-U6-rbcS:GFP (V111). For expression of the fluorescent marker in roots we amplified a 35S promoter devoid of BbsI sites from pICH51266 (a gift from Sylvestre Marillonnet and Nicola Patron (Addgene plasmid # 50267)) with attached SbfI and PacI sites (using primer 35S-SbfI-sense and 35S-PacI-anti) and ligated it with the PacI/SbfI restricted vector resulting in pB-CRISPR+35S::GFP (V112).

For the complementation, *BcFLA1a_mut_* was produced synthetically by IDT (Integrated DNA Technologies, Inc., Coralville, IA, USA) and contained a ClaI restriction site at the beginning and a PstI restriction site at the end for the insertion into the MCS of V69 vector resulting in H279. Then the overexpression cassette from H279 was cut by AscI and PmeI and, after blunting the AscI digested end, inserted into the CRISPR+35S::GFP+fla1-guides (H278), which was digested by PmeI, resulting in H280.

Fauser F, Roth N, Pacher M, Ilg G, Sanchez-Fernandez R, Biesgen C, et al. In planta gene targeting. Proc Natl Acad Sci U S A. 2012; 109: 7535–7540. doi: 10.1073/pnas.1202191109.

Myrach T, Zhu A, Witte C-P. The assembly of the plant urease activation complex and the essential role of the urease accessory protein G (UreG) in delivery of nickel to urease. J Biol Chem. 2017. doi: 10.1074/jbc.M117.780403.

Feng Z, Zhang B, Ding W, Liu X, Yang D-L, Wei P, et al. Efficient genome editing in plants using a CRISPR/Cas system. Cell Res. 2013; 23: 1229–1232. doi: 10.1038/cr.2013.114.

Mao Y, Zhang H, Xu N, Zhang B, Gou F, Zhu J-K. Application of the CRISPR-Cas system for efficient genome engineering in plants. Mol Plant. 2013; 6: 2008–2011. doi: 10.1093/mp/sst121.

Dadami E, Moser M, Zwiebel M, Krczal G, Wassenegger M, Dalakouras A. An endogene-resembling transgene delays the onset of silencing and limits siRNA accumulation. FEBS Lett. 2013; 587: 706–710. doi: 10.1016/j.febslet.2013.01.045.
