# Supplementary material for: Efficient generation of mutations mediated by CRISPR/Cas9 in the hairy root transformation system of Brassica carinata
Source: PLoS One. 2017 Sep 22;12(9):e0185429. doi: 10.1371/journal.pone.0185429 (PMC5609758; doi:10.1371/journal.pone.0185429)
Supplement: S2 Text — (DOCX) [file pone.0185429.s007.docx]

**S2 Text.** **Expression analysis.**

cDNA synthesis was performed using the ‘PrimeScript™ 1st strand cDNA Synthesis Kit’ (Takara Bio Europe SAS, Saint-Germain-en-Laye, France) according to the manufacturer’s instructions. 1 µg of the isolated RNA was applied in a 20 µL reaction. The cDNA was diluted 1:1 with H_2_O, quantified (NanoPhotometer^®^ P-Class P 300, Implen, Munich, Germany) and stored at -85°C.

Apart from the endogenous control gene *UBIQUITIN CONJUGATING ENZYME 9* (*AtUBC9*; AT4G27960) (Czechowski *et al*., 2005), qPCR primers were designed using Primer3Plus (Untergasser *et al*., 2012) (S2 Table) in consideration of the following quality standards: amplicon size between 90 and 200 bp, primer size between 20 and 25 bp, primer T_m_ between 58 and 62°C and a GC content between 40 and 60 %. The specific primers were designed on the two gRNA regions with three differing bases at the 3’ end of the primers, whereas the binding sites for the unspecific primer pair is located at the 3’ end of *BcFLA1*. Confirmation of the primer specificity and the pretest were carried out by PCR using the ‘DCS DNA HotStart Polymerase’ (DNA Cloning Service e.K., Hamburg, Germany) according to the manufacturer’s instructions and a primer concentration of 0.25 µM. The amplicons were separated on a 4 % (w/v) non-denaturating agarose gel containing 0.004 % (v/v) Midori Green Advance (Nippon Genetics Europe, Dueren, Germany) for 35-40 min at 7.6 V cm^-1^ in TAE buffer.

Besides *AtUBC9* (Czechowski *et al*., 2005), qPCR was performed using a second endogenous control, *ELGONGATION FACTOR 1 ALPHA 1* (*BcEF-1-a1*), which exhibited a uniform expression in the MACE dataset. The partial cDNA sequence of *BcEF-1-alpha1* obtained by MACE analysis, was submitted to the NCBI database under accession number MF061227.

For primer efficiency tests, qPCR was performed using seven different cDNA amounts per reaction (200, 40, 8, 1.6, 0.32, 0.064, 0.0128 ng cDNA), whereas consistently 100 ng cDNA per reaction were used for expression analysis. Only primer pairs with an efficiency between 90 and 110 % were used.

Three technical replications were performed per transgenic root. The components per 10 µL reaction were as follows: 5 µL 2x ‘SYBR® Premix Ex Taq™ (Tli RNaseH Plus)’ (Takara Bio Europe SAS, Saint-Germain-en-Laye, France), 0.25 µM forward and reverse Primer, 0.5 µL H_2_O and 4 µL of the diluted cDNA.

The quantitative reverse transcriptase polymerase chain reaction (qPCR) runs were performed in the CFX96 C1000 Thermal cycler (Bio-Rad, Hercules, CA, USA) with an initial step of 95 °C for 30 s, followed by 40 cycles of 95 °C for 5 s and 60 °C or 65°C for 30 s as well as a closing step of 95°C for 10 s and a final melting curve procedure with a stepwise increment of 0.5 °C ranging from 65 °C to 95 °C.

Czechowski T, Stitt M, Altmann T, Udvardi MK, Scheible W-R. Genome-wide identification and testing of superior reference genes for transcript normalization in Arabidopsis. Plant Physiol. 2005; 139: 5–17. doi: 10.1104/pp.105.063743.

Untergasser A, Cutcutache I, Koressaar T, Ye J, Faircloth BC, Remm M, et al. Primer3--new capabilities and interfaces. Nucleic Acids Res. 2012; 40: e115. doi: 10.1093/nar/gks596.
