# Supplementary material for: Efficient generation of mutations mediated by CRISPR/Cas9 in the hairy root transformation system of Brassica carinata
Source: PLoS One. 2017 Sep 22;12(9):e0185429. doi: 10.1371/journal.pone.0185429 (PMC5609758; doi:10.1371/journal.pone.0185429)
Supplement: S1 Table — (DOCX) [file pone.0185429.s008.docx]

**S1 Table. Primers used for construct preparation.**

| **Oligo name** | **Primer sequence forward / reverse (5’🡪 3’)** |
| --- | --- |
| gRNA1-F | TAGGTCTCCAGAGGAAGACAGGTTTTAGAGCTAGAA |
| gRNA1-R | ATGGTCTCACTCTCCACGGAGTGCACCAGCCGGGAA |
| gRNA2-F | TAGGTCTCCTCGCCGTGATGTGTTTTAGAGCTAGAA |
| gRNA2-R | ATGGTCTCAGCGATCCTTGAATGCACCAGCCGGGAA |
| L5AD5-F | CGGGTCTCAGGCAGAAGACTAGATTGAACAAAGCACCAGTGG |
| L5AD5-R | TAGGTCTCCAAACGAAGACAAAAACAAAAAAAAAAGCACCGACTCG |
| S5AD5-F | CGGGTCTCAGGCAGAAGACTAGATT |
| S5AD5-R | TAGGTCTCCAAACGAAGACAAAAAC |
| Ubi-sense | TATGGCGCGCCAAAAATTACGGATATGAATATAGGC |
| Ubi-anti | ATACTCGAGGCTGCACATACATAACATATCA |
| Cas9-sense | TTATGAATTCATGGACTATAAGGACCAC |
| Cas9-anti | CTATCCCGGGTTACTTTTTCTTTTTTGC |
| guide-RNA-sense | TATCGGGTACCGAGCTCGGATCCA |
| guide-RNA-anti | TATCTACGCGTCTCGAGCGGCCGCCAGTG |
| MCS1 | GGCCGGACGTCCCTTAGGTACCCTACACGCGTCTAGACCGGTGTTT |
| MCS2 | AAACACCGGTCTAGACGCGTGTAGGGTACCTAAGGGACGTCC |
| GFP-1 | tttcccgccttcggtttgggcctgcaggTTAGGGCACGATGTCCTG |
| GFP-2 | gaaaaagattAGTTTTCATTGTTAATTAAACTTAGACAG |
| GFP-3 | aatgaaaactAATCTTTTTCTCTTTCTCATCTTTTC |
| GFP-4 | taaatatgtcGTCATCTTCTTCTTTTTTTTTTGG |
| GFP-5 | agaagatgacGACATATTTATATATTTAGAAAAAATTTAACTTTTAAC |
| GFP-6 | gttgcacgccGCCATCTTCGATGTTGTG |
| GFP-7 | cgaagatggcGGCGTGCAACTCGCTGAT |
| GFP-8 | ttcatatccgtaatttttggCCGCTCTAGAAGTGGTTGG |
| 35S-SbfI-sense | AACCTGCAGGGAATTCCAATCCCACAAAAATCTGAGCTTA |
| 35S-PacI-anti | AATTAATTAAATCGATAATTGTAAATGTAA |
