# Supplementary material for: Efficient generation of mutations mediated by CRISPR/Cas9 in the hairy root transformation system of Brassica carinata
Source: PLoS One. 2017 Sep 22;12(9):e0185429. doi: 10.1371/journal.pone.0185429 (PMC5609758; doi:10.1371/journal.pone.0185429)
Supplement: S2 Table — Wildtype and mutant-specific primers were designed on the two gRNA regions harboring three differing bases at the 3’ end (underlined). The unspecific primers are located at the 3’ end of BcFLA1. (DOCX) [file pone.0185429.s009.docx]

**S2 Table. qPCR primers.**

| **Description** | **Sequence forward / reverse**  **(5’ 🡪 3’)** | **T_A_ [°C]** |
| --- | --- | --- |
| *AtUBC9* | TCACAATTTCCAAGGTGCTGC  TCATCTGGGTTTGGATCCGT | 65 |
| *BcEF-1-alpha* | GGTGACGCTGGTATGGTGAA  TGATGACACCGACTGCAACA | 60 |
| *BcFLA1* (unspec. qPCR) | TCGTTGAAACACGTGTCAGT  ACTTATCCGATGGAGCCCCT | 65 |
| *BcFLA1a_mut_* (qPCR) | CTCATCCTTGCTGCTGTGTTT  CAAGGATCGCCGTAATATTAGTCG | 65 |
| *BcFLA1* (wildtype-spec. qPCR) | TCATCCTTGCCGCTGTCTTC  CAAGGATCGCCGTGATGTTG | 65 |

Wildtype and mutant-specific primers were designed on the two gRNA regions harboring three differing bases at the 3’ end (underlined). The unspecific primers are located at the 3’ end of BcFLA1.
