# Supplementary material for: Efficient generation of mutations mediated by CRISPR/Cas9 in the hairy root transformation system of Brassica carinata
Source: PLoS One. 2017 Sep 22;12(9):e0185429. doi: 10.1371/journal.pone.0185429 (PMC5609758; doi:10.1371/journal.pone.0185429)
Supplement: S3 Table — For M13 SSR-PCR forward primers were tagged with M13 tail GTAAAACGACGGCCAGT. Numbers in brackets indicate amplicon size including the M13 tail. (DOCX) [file pone.0185429.s010.docx]

**S3 Table. Primers for the verification of the gene editing.**

| **Description** | **Sequence forward / reverse**  **(5’ 🡪 3’)** | **T_A_ [°C]** | **Amplicon size [bp]** |
| --- | --- | --- | --- |
| *BcFLA1* (unspec. large) | TTCAAACGCAATGGCAACCA  ACCCAAATCAAACGACGAGTTTC | 60 | 752 (769) |
| *BcFLA1a_mut_* (large) | TGCGGCGCCATTAAATAACG  TTGAGTCCTTACCGGGTTACTT | 60 | 713 (730) |
| *BcFLA1* (wildtype-spec. large) | AACTCAAAACTTCAAACGCAATGGC  GAAACCCAAATCAAACGACGAGT | 63 | 665 (682) |
| *BcFLA1* (unspec. small) | TTCAAACGCAATGGCAACCA  TTCCGACTCGTGTTGTGTTT | 60 | 200 (217) |
| *BcFLA1a_mut_* (small) | CCCGTGGTTTTCGAACTTGG  TTCCGACTCGTGTTGTGTTT | 60 | 237 (254) |
| *BcFLA1* (wildtype-spec. small) | AACTCAAAACTTCAAACGCAATGGC  TTCCGACTCGTGTTGTGTTT | 63 | 210 (227) |

For M13 SSR-PCR forward primers were tagged with M13 tail GTAAAACGACGGCCAGT. Numbers in brackets indicate amplicon size including the M13 tail.
